# Causal effects of circulating lipids and lipid-lowering drugs on the risk of urinary stones: a Mendelian randomization study

**DOI:** 10.3389/fendo.2023.1301163

**Published:** 2023-12-01

**Authors:** Zilong Tan, Jing Hong, Aochuan Sun, Mengdi Ding, Jianwu Shen

**Affiliations:** ^1^ Department of Urology, Xiyuan Hospital, China Academy of Chinese Medical Sciences, Beijing, China; ^2^ School of Basic Medical Sciences, Peking University, Beijing, China; ^3^ Department of Geriatrics, Xiyuan Hospital, China Academy of Chinese Medical Sciences, Beijing, China

**Keywords:** circulating lipids, lipid-lowering drugs, urinary stones, Mendelian randomization, causality, genetics

## Abstract

**Background:**

Previous studies have yielded conflicting findings regarding the association between circulating lipids and lipid-lowering drugs with urinary stones, and the causal relationship between the two remains inconclusive.

**Objective:**

This study aimed to assess the causal relationship between circulating lipids (Triglycerides [TG], low-density lipoprotein cholesterol [LDL-C], high-density lipoprotein cholesterol [HDL-C], apolipoprotein A [APOA], apolipoprotein B [APOB] and Pure hypercholesterolaemia), lipid-lowering drugs (HMGCR [HMG-CoA reductase] inhibitors and PCSK9[Proprotein Convertase Subtilisin/Kexin Type 9] inhibitors) and the risk of urinary stones, using genetic data.

**Methods:**

Genetic instrumental variables (GIVs) for circulating lipids and lipid-lowering drugs were obtained from the UK Biobank and existing literature. Outcome data were extracted from a genetic association database with 3,625 urinary stone cases and 459,308 controls. Two-sample MR analysis, employing the TwoSampleMR software package in R 4.2.3, was conducted to assess the associations between multiple exposures. The primary outcome was determined using the inverse variance weighted (IVW) method for the causal relationship between exposure and outcome, while additional methods such as MR-Egger, weighted median, simple mode, and weighted mode were utilized as supplementary analyses. Robustness of the Mendelian Randomization (MR) analysis results was assessed through leave-one-out analysis and funnel plots.

**Results:**

The MR analysis revealed a significant association between elevated TG levels per 1 standard deviation and the occurrence of urinary stones (odds ratio [OR]: 1.002, 95% confidence interval [CI]: 1.000-1.003, P = 0.010). However, no significant association was observed between factors other than TG exposure and the risk of urinary stone occurrence across all methods(LDL-C: [OR], 1.001; 95% [CI], 1.000-1.003, P=0.132;HDL-C: [OR], 0.999; 95% [CI], 0.998-1.000, P=0.151;APOA:[OR] being 1.000 (95% [CI], 0.999-1.001, P=0.721;APOB: [OR] of 1.001 (95% [CI], 1.000-1.002, P=0.058;Pure hypercholesterolaemia: [OR] of 1.015 (95% [CI], 0.976-1.055, P=0.455) and lipid-lowering drugs (HMGCR inhibitors: [OR], 0.997; 95% [CI], 0.990-1.003, P=0.301 and PCSK9 inhibitors:[OR], 1.002; 95% [CI], 1.000-1.005, P=0.099).

**Conclusion:**

Our findings provide conclusive evidence supporting a causal relationship between an increased risk of urinary stones and elevated serum TG levels. However, we did not find a significant association between urinary stone occurrence and the levels of LDL-C, HDL-C, APOA, APOB, Pure hypercholesterolaemia and lipid-lowering drugs.

## 1 Introduction

Urinary stones represent a prevalent condition in urology, with a reported overall prevalence of 11.0% in certain populations (1). The occurrence of urinary stones tends to increase with age in both sexes, reaching a peak prevalence of approximately 19.4% within the 60-69 year age range (2). Recent epidemiological studies conducted in China have estimated a prevalence of kidney stones at approximately 5.8%, with a substantial proportion of patients (30%-50%) being at risk of recurrent stones within a decade (3–5). The development of urinary stones is thought to be influenced by various factors, including genetics, age, gender, and underlying medical conditions (5–8). Consequently, understanding the etiology and risk factors associated with urinary stones has become an important research focus, providing valuable insights for the disease prevention and treatment strategies (9).

Circulating lipids encompass a group of essential substances in the blood, including TG, LDL-C, HDL-C, APOA, APOB, cholesterol, and other metabolically important compounds. However, disruptions in lipid metabolism, as common metabolic disorders, have been linked to the onset and advancement of various illnesses that impact multiple bodily systems (10–13). In recent years, the relationship between metabolic disorders and the prevalence of urinary stones has gained attention, suggesting a potential link between circulating lipids and the occurrence of urinary stones (14–16). Furthermore, lipid-lowering drugs, particularly statins, have shown promise in reducing the risk of such disorder (17–19). Previous research has indicated that the modulation of lipid equilibrium via the administration of lipid-lowering medications, notably HMGCR inhibitors targeting HMG-CoA reductase-an essential enzyme in cholesterol synthesis—can impact serum LDL-C levels (20, 21). Additionally, PCSK9 inhibitors, targeting proprotein convertase subtilisin/kexin type 9, have been shown to decrease triglyceride, APOA, APOB, and LDL-C levels while increasing HDL-C levels (22–24). Numerous international studies have investigated whether hypertriglyceridemia serves as a risk factor for urinary stones. However, the findings across these studies remain contentious. This ongoing controversy can likely be attributed to variations in genetic backgrounds, gender distributions, age demographics, and the geographical locations of the populations under investigation (25–27). This study aims to investigate not only the association between TG in the circulatory system and urinary stones but also includes other lipid-related substances and lipid-lowering drugs as exposure factors. By maximizing suitable sample sizes, this study seeks to address potential limitations of previous research, provide comprehensive insights into the associations between multiple exposure factors and urinary stone occurrence, and furnish evidence-based medical information for clinical diagnosis, treatment, and prevention.

In general, the most robust and dependable experimental approach for investigating causal relationships between risk factors and outcome events is the randomized controlled trial (RCT). However, in etiological studies, the implementation of RCTs can be challenging due to ethical constraints and various other limitations. Large-scale RCTs exploring the association between circulating lipids, lipid-lowering drugs, and the risk of urinary stones are notably absent. Moreover, it is imperative to acknowledge the difficulty observational studies face in mitigating potential confounding biases between exposures and outcomes (28). As a result, the causal connection between circulating lipids, lipid-lowering drugs, and the risk of urinary stones remains uncertain. Mendelian randomization, an innovative analytical approach rooted in genetic information, employs random genetic variants, particularly single nucleotide polymorphisms (SNPs), as instrumental variables to mimic the random allocation inherent in RCTs, prior to the occurrence of the outcome event (29). Consequently, MR analysis is relatively impervious to confounders and serves as a dependable tool for assessing the causal impact of risk factor exposure on outcomes (30–32). The objective of our study was to investigate, through MR analysis based on extensive cohort GWAS data, the causal relationship between six major lipid metabolism-related substances, two lipid-lowering drug targets of action, and the occurrence of urinary stones.

## 2 Methods

### 2.1 Study design and data sources

**Figure 1:**
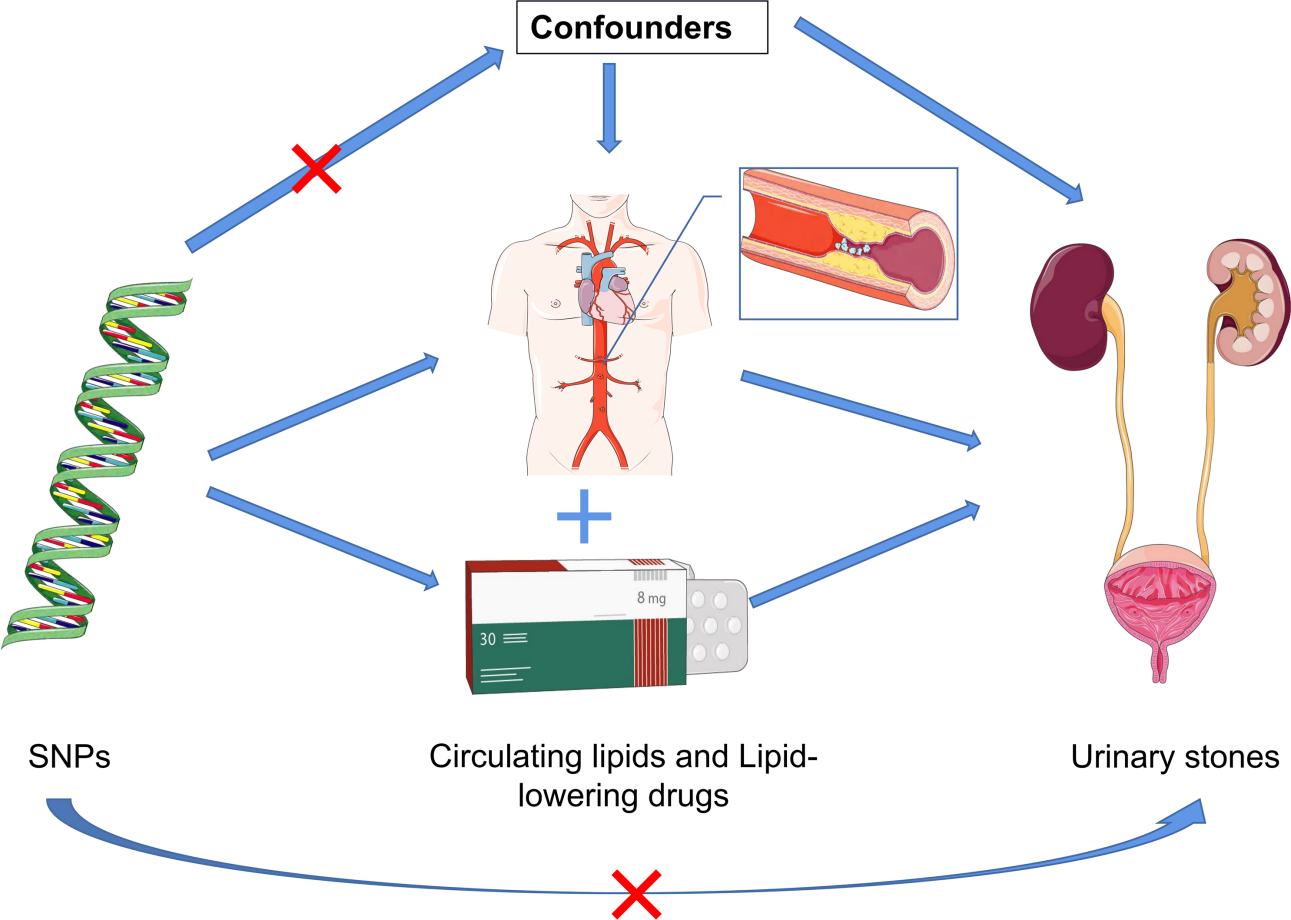
Schematic representation of the three assumptions and study design. (1) The employed genetic IVs are firmly linked to the exposure; (2) The chosen IVs exhibit no associations with potential confounding factors; (3) The IVs can solely influence the outcome risk through the exposure in a dependent manner.

To obtain comprehensive and robust insights into the causal relationship between circulating lipids and lipid-lowering drugs concerning urinary stones, we employed a two-sample MR analysis model **Figure 1**. Genetic variants associated with circulating lipids were sourced from the recent UK Biobank GWAS based on UK Biobank data (33, 34). Specifically, the serum lipid GWAS with the most substantial sample size to date was selected from the UK Biobank data, including 441,016 patients for triglycerides (TG, ieu-b-111), 440,456 patients for low-density lipoprotein cholesterol (LDL-C, ieu-b-110), 403,943 patients for high-density lipoprotein cholesterol (HDL-C, ieu-b-109), 393,193 patients for apolipoprotein A (APOA, ieu-b-107), 439,214 patients for apolipoprotein B (APOB, ieu-b-108), and 463,010 patients for Pure hypercholesterolaemia (ieu-b-108, expressed as a dichotomous variable).These lipid measurements (TG, LDL-C, HDL-C, APOA, and APOB) in our study adhered to uniform standard serum lipid and apolipoprotein assay protocols, as they originated from the same clinical trial. Consequently, the results are expressed as continuous variables, allowing us to use standard deviations (SD) as the unit for assessing the magnitude of changes in circulating lipids concerning outcome events (Pure hypercholesterolemia was presented as a binary variable.). Summary statistics for genetic variants associated with lipid-lowering drug targets were derived from a recently published study (35). The summary statistics employed in our study, focusing on urinary stones as the outcome, were obtained from a GWAS analysis based on individuals of European ancestry, comprising 462,933 patients (ID: ukb-b-8297, https://gwas.mrcieu.ac.uk/datasets/ukb-b-8297/). This analysis encompassed 3,625 cases of urinary stones and 459,308 control subjects, with the final GWAS yielding up to 9,851,867 associations between genotypic SNPs and urinary stones (**Table 1**). It is noteworthy that all studies contributing to this GWAS meta-analysis received approval from relevant institutional review boards. Importantly, our present study did not necessitate separate ethical approval, the ethical approval and consent information for the above summary statistics were taken from the original publication.

**Table 1:** Baseline characteristics of lipids, lipid-lowering drugs, and urinary stones.

Trait	ID	Year	Consortium	Population/Sex	Sample Size	n Case	n Control	n SNPs
**TG**	**ieu-b-111**	**2020**	**UK Biobank**	**European/Males and Females**	**441,016**			**12,321,875**
**LDL-C**	**ieu-b-110**	**2020**	**UK Biobank**	**European/Males and Females**	**440,546**			**12,321,875**
**HDL-C**	**ieu-b-109**	**2020**	**UK Biobank**	**European/Males and Females**	**403,943**			**12,321,875**
**APOB**	**ieu-b-108**	**2020**	**UK Biobank**	**European/Males and Females**	**439,214**			**12,321,875**
**APOA**	**ieu-b-107**	**2020**	**UK Biobank**	**European/Males and Females**	**393,193**			**12,321,875**
**Pure hypercholesterolaemia**	**ukb-b-12651**	**2018**	**MRC-IEU**	**European/Males and Females**	**463,010**	**22,622**	**440,388**	**9,851,867**
**HMGCR inhibitors**	https://elifesciences.org/articles/73873							
**PCSK9 inhibitors**								
**kidney/ureter/bladder stone**	**ukb-b-8297**	**2018**	**MRC-IEU**	**European/Males and Females**	**462,933**	**3,625**	**459,308**	**9,851,867**

TG, triglycerides; LDL-C, low-density lipoprotein cholesterol; HDL-C, high-density lipoprotein cholesterol; APOA, Apolipoprotein A; APOB, Apolipoprotein B; HMGCR, HMG-CoA reductase; PCSK9, Proprotein Convertase Subtilisin/Kexin Type 9.

### 2.2 SNPs selection

We have successfully identified independent SNPs associated with plasma levels of TG, LDL-C, HDL-C, APOA, APOB, and Pure hypercholesterolaemia based on three fundamental hypotheses. Firstly, we initiated the selection process by identifying autosomal bi-allelic SNPs with a significance level of P<5e^-8^. To avoid potential confusion, we investigated each instrumental SNP (36, 37) in the PhenoScanner GWAS database to assess any previous associations (P<5e^-8^) with plausible confounders (i.e., age, gender, diet, high uric acid levels and type 2 diabetes.) (38, 39). In order to adhere to the assumption that only instruments exclusively related to the exposure are related to the outcome, we excluded SNPs highly correlated with the outcome.To ensure comparability of effect estimates across lipid-associated traits in the UK Biobank, we normalized these traits using inverse rank normalization. It’s worth noting that this approach facilitates the comparison of effect estimates among different traits. For the outcome event, which is the occurrence of urinary stones, it was treated as a dichotomous event and did not necessitate specific transformation. Secondly, to establish the independence of the selected genetic variants, we systematically removed SNPs that exhibited linkage disequilibrium (r^2^ > 0.01) and were situated in close proximity within a 1000-kilobase aggregation window of other SNPs with higher P-values. Lastly, we employed an F-statistics threshold greater than 10 to identify robust instrumental variables, thus mitigating the potential effects of bias. It is important to note that our SNPs screening approach aligned with methodologies utilized in previously published studies, ensuring consistency and reliability in our selection of genetic variants associated with two lipid-lowering drug gene targets and their respective SNP datasets.

### 2.3 SNP final selection

**Figure 2:**
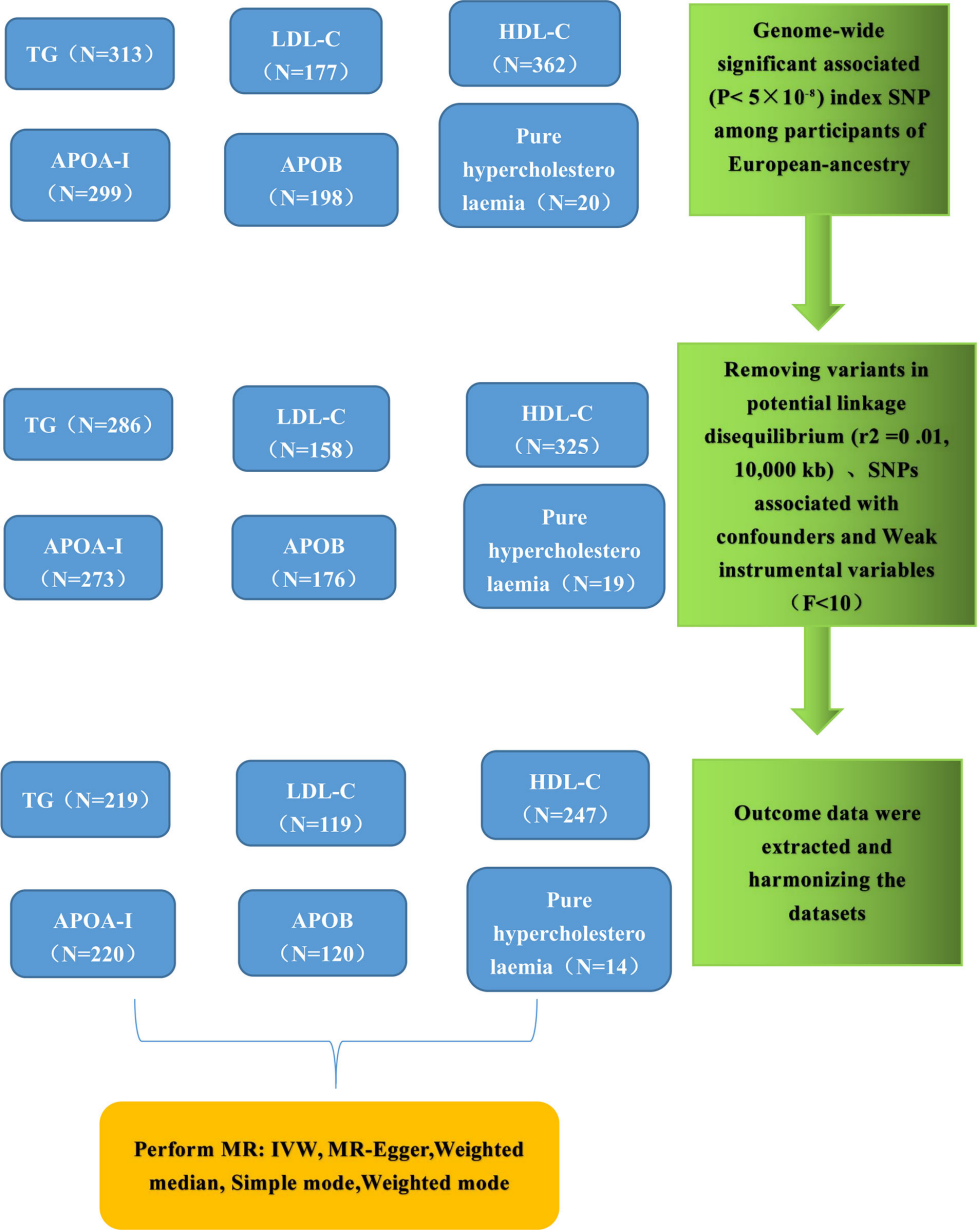
Genetic instrument selection of single-variable Mendelian randomization study. TG, Triglycerides; LDL-C, low-density lipoprotein cholesterol; HDL-C, high-density lipoprotein cholesterol; APOA, apolipoprotein; APOB, apolipoprotein; IVW, inverse-variance weighting; MR, Mendelian randomization; SNP, single nucleotide polymorphisms.

The flowchart illustrating the significant SNP screening process, along with the exposures and outcomes, is presented in **Figure 2**. Following the stipulated criteria, we identified a total of 286 SNPs for TG, 158 SNPs for LDL-C, 325 SNPs for HDL-C, 273 SNPs for APOA, 176 SNPs for APOB, 19 SNPs for Pure hypercholesterolaemia, and 7 and 8 SNPs for HMGCR inhibitors and PCSK9 inhibitors, respectively, within the UK Biobank (UKB) dataset. Subsequently, we meticulously curated and harmonized this SNP dataset, resulting in the inclusion of 219, 119, 247, 220, 120, 14, 7 and 8 SNPs for TG, LDL-C, HDL-C, APOA, APOB, Pure hypercholesterolaemia, HMGCR inhibitors and PCSK9 inhibitors, respectively, for use in the Mendelian randomization (MR) analysis. All the SNPs employed in this MR analysis are listed comprehensively in **Supplementary Tables S1-S8**.

### 2.4 Mendelian randomization analysis

The two-sample Mendelian randomization analysis was executed using TwoSampleMR version 0.5.7 (https://github.com/MRCIEU/TwoSampleMR) within the R 4.2.3 environment. To evaluate causality, we employed a total of five MR analysis methods, encompassing the IVW, MR Egger, Weighted Median, Simple Mode, and Weighted Mode approaches. Our primary method of assessment was the IVW method, which assigns weights to each ratio based on their standard errors (SE) while accounting for potential heterogeneity in measurements. This method yields reliable causal estimates even in the presence of heterogeneity. Since all instrumental variables must conform to MR assumptions in the IVW method, we also employed two other methods, weighted median estimation, and MR-Egger, for sensitivity analysis. The weighted median estimation method provides a consistent assessment of causality when more than half of the instrumental variables are deemed valid. Furthermore, we employed the IVW method to gauge the pleiotropy and heterogeneity of individual SNPs (40). Additionally, MR-Egger regression was conducted in this study, enabling the detection and adjustment of pleiotropy, thereby yielding an assessment of causal effects (41). It aids in determining whether directional-level pleiotropy, rather than exposure directly affecting the outcome, underlies the observed results through other pathways. Furthermore, we conducted leave-one-out analyses to assess the robustness of our MR results in the presence of any outlier SNPs. In this MR study, we considered three criteria for assuming causality:(1) A P-value for IVW < 0.05. Consistency in the direction of estimation between the IVW method, the MR-Egger method, and the weighted median method. (2)A P-value > 0.05 for the MR-Egger intercept test considered statistically significant.

## 3 Results

### 3.1 Causal effects of circulating lipids on the risk of urinary stones via MR

**Figure 3:**
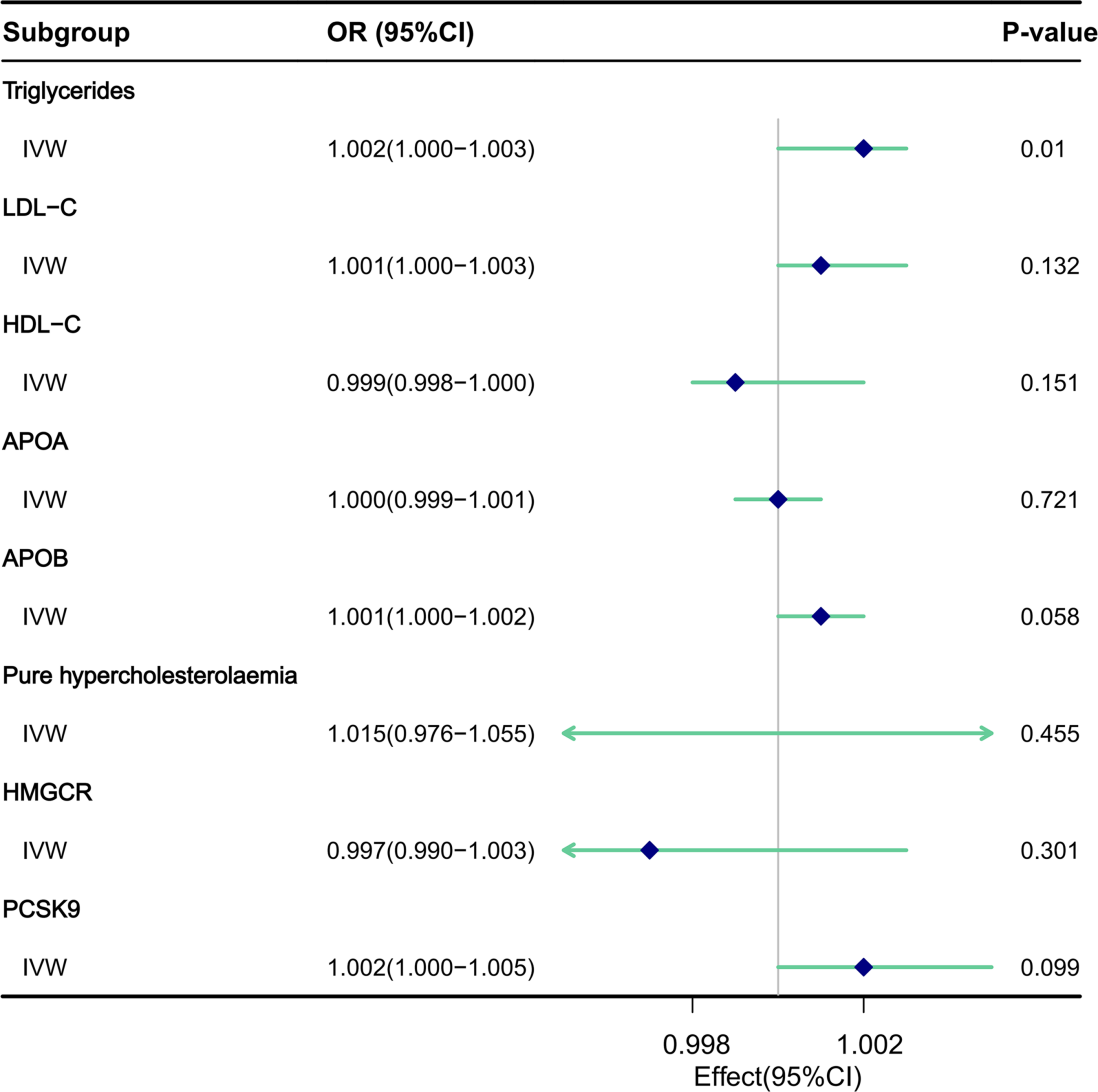
The impact of circulating lipids and lipid-lowering drugs on the risk of urinary stones was assessed through MR analysis utilizing the IVW model.

IVW MR method was utilized to analyze the final results (**Figure 3**). In addition to IVW, other MR analysis methods, including MR Egger, Simple Mode, Weighted Mode, and Weighted Median, were employed to complement IVW, thereby reinforcing the robustness of the IVW analysis outcomes. The IVW analysis, depicted in **Supplementary Figure 5A**, demonstrated a causal relationship between TG and an elevated risk of urinary stones, yielding a significant difference (Odds Ratio [OR], 1.002; 95% Confidence Interval [CI], 1.000-1.003, P=0.010). Consistent results were observed across other analytical methods, as detailed in **Supplementary Table 1** and **Supplementary Figure 4A**. Notably, heterogeneity was detected in the IVW analysis (Q=284.035, P=0.00036). MR-Egger regression analysis revealed no evidence of directional pleiotropic effects among the genetic variants (P=0.524) (**Table 2**). Furthermore, the results of leave-one-out sensitivity analyses indicated that the association between triglycerides and urinary stone disease was not substantially driven by any single SNP, as illustrated in **Supplementary Figure 6A**. Funnel plots, as presented in **Supplementary Figure 7A**, underscored the stability of our analytical approach.

**Table 2:** Tests of pleiotropy of selected SNPs and heterogeneity between SNPs.

Exposure	Pleiotropy Test		Heterogeneity Test	
	Beta (SE)	P Value	Cochran’s Q	P Value
**Triglycerides**	2.907e-05	0.524	284.035	0.00036
**LDL-C**	3.730e-05	0.128	150.751	0.012
**HDL-C**	2.679e-05	0.169	300.182	0.0005
**Apolipoprotein A**	2.910e-05	0.634	324.343	2.040e-06
**Apolipoprotein B**	3.785e-05	0.239	167.572	0.002
**Pure hypercholesterolaemia**	0.00025	0.842	24.086	0.012
**HMGCR inhibitors**	0.001	0.937	26.647	1.685e-04
**PCSK9 inhibitors**	0.00029	0.251	4.335	0.740

MR analysis utilizing the IVW model (**Supplementary Figure 5B**) did not reveal any association between LDL-C and an increased risk of urinary stones, with no statistically significant difference ([OR], 1.001; 95% [CI], 1.000-1.003, P=0.132). Consistent results were obtained when employing other analytical methods, as detailed in **Supplementary Table 2** and **Supplementary Figure 4B**. It is noteworthy that evidence of heterogeneity emerged in the IVW analysis (Q=150.751, P=0.012). MR-Egger regression analysis provided no indication of a directed pleiotropic effect within the genetic variance (P=0.128) (**Table 2**). Furthermore, the results of the leave-one-out sensitivity analysis demonstrated that the association between LDL cholesterol and urinary stone disease was not primarily driven by any individual SNP, as illustrated in **Supplementary Figure 6B**. Additionally, the funnel plots, as shown in **Supplementary Figure 7B**, emphasized the relative stability of our analytical approach.

MR analysis utilizing the IVW model (**Supplementary Figure 5C**) revealed no causal link between HDL-C and a reduced risk of urinary stones, with no significant difference ([OR], 0.999; 95% [CI], 0.998-1.000, P=0.151). These findings remained consistent across other analytical methods, as reported in **Supplementary Table 3** and **Supplementary Figure 4C**. It is notable that heterogeneity was evident in the IVW analysis (Q=300.182, P=0.0005). MR-Egger regression analysis detected no directional pleiotropic effect within the genetic variance (P=0.169) (**Table 2**). Moreover, leave-one-out sensitivity analyses demonstrated that the association between HDL cholesterol and urinary stone disease was not primarily influenced by any single SNP, as depicted in **Supplementary Figure 6C**. The funnel plots, illustrated in **Supplementary Figure 7C**, underscored the relative stability of our interpretation.

MR analysis utilizing the IVW model (**Supplementary Figure 5D**) did not reveal any causal association between APOA and an increased risk of urinary stones, with the [OR] being 1.000 (95% [CI], 0.999-1.001, P=0.721), as detailed in **Table 1**. Consistent findings were obtained when employing other analytical methods, as presented in **Supplementary Table 4** and **Supplementary Figure 4D**. Significantly, the IVW analysis uncovered evidence of heterogeneity (Q=324.343, P=2.040e-06). MR-Egger regression analysis provided no indication of pleiotropic effects within the genetic variance (P=0.634) (**Table 2**). Furthermore, leave-one-out sensitivity analyses demonstrated that the association between APOA and urinary stones was not predominantly driven by any single SNP, as illustrated in **Supplementary Figure 6D**. The funnel plot, depicted in **Supplementary Figure 7D**, underscored the stability of the results we obtained.

MR analysis utilizing the IVW model (**Supplementary Figure 5E**) revealed that APOB was not causally associated with an increased risk of urinary stones, with the [OR] of 1.001 (95% [CI], 1.000-1.002, P=0.058), as detailed in **Table 1**. Consistent findings were obtained when employing other analytical methods, as presented in **Supplementary Table 4** and **Supplementary Figure 4E**. Notably, the IVW analysis identified evidence of heterogeneity (Q=167.572, P=0.002). MR-Egger regression analysis indicated no evidence of a pleiotropic effect (P=0.239) (**Table 2**). Furthermore, leave-one-out sensitivity analyses demonstrated that the association between APOB and urinary stone disease was not primarily influenced by any individual SNP, as illustrated in **Supplementary Figure 6E**. Funnel plots, as depicted in **Supplementary Figure 7E**, underscored the relative stability of our interpretation.

MR analysis utilizing the IVW model (**Supplementary Figure 5F**) revealed no causal association between Pure hypercholesterolaemia and an increased risk of urinary stones, with the [OR] of 1.015 (95% [CI], 0.976-1.055, P=0.455), as detailed in **Table 1**. Similar results were observed across other analytical methods, as reported in **Supplementary Table 6** and **Supplementary Figure 4F**. Notably, heterogeneity was observed in the IVW analysis (Q=24.086, P=0.012). MR-Egger regression analysis found no evidence of directed pleiotropic effects in genetic variants (P=0.842) (**Table 2**). Furthermore, leave-one-out sensitivity analyses indicated that the association between cholesterol and urinary stone disease was not primarily driven by any individual SNP, as demonstrated in **Supplementary Figure 6F**. The funnel plot, as presented in **Supplementary Figure 7F**, also revealed no aberrations in our interpretation.

### 3.2 Causal effects of lipid-lowering drugs on the risk of urinary stones via MR

The impact of lipid-lowering drugs on the risk of urinary stones was assessed through MR analysis utilizing the IVW model (**Supplementary Figure 5G**). The analysis did not reveal any causal relationship between HMGCR inhibitors and a reduced risk of urinary stones, with no statistically significant difference observed ([OR], 0.997; 95% [CI], 0.990-1.003, P=0.301). Consistent findings were observed when employing other analytical methods, as detailed in **Supplementary Table 7** and **Supplementary Figure 4G**. It is noteworthy that evidence of heterogeneity was present in the IVW analysis (Q=26.647, P=1.685e-04). MR-Egger regression analysis showed no indication of directional pleiotropy within the genetic variance (P=0.937) (**Table 2**). Moreover, the results of the leave-one-out sensitivity analysis indicated that the association between HMGCR inhibitors and urinary stone disease was not predominantly influenced by any single SNP, as illustrated in **Supplementary Figure 6G**. Additionally, the funnel plot, as presented in **Supplementary Figure 7G**, emphasized the relative stability of our interpretation.

Similarly, MR analysis utilizing the IVW model (**Supplementary Figure 5H**) did not reveal any causal association between PCSK9 inhibitors and a reduced risk of urinary stones, with no statistically significant difference ([OR], 1.002; 95% [CI], 1.000-1.005, P=0.099). These findings remained consistent across other analytical methods, as reported in **Supplementary Table 8** and **Supplementary Figure 4H**. Notably, evidence of heterogeneity was present in the IVW analysis (Q=4.335, P=0.740). MR-Egger regression analysis detected no indication of a directed pleiotropic effect within the genetic variance (P=0.251) (**Table 2**). Furthermore, the results of the leave-one-out sensitivity analysis demonstrated that the association between PCSK9 inhibitors and urinary stone disease was not predominantly driven by any single SNP, as depicted in **Supplementary Figure 6H**. The funnel plots, as illustrated in **Supplementary Figure 7H**, underscored the stability of our analytical approach.

To counter the potential impact of reverse causality, we conducted a reverse Mendelian randomization (MR) analysis to validate our study. This approach examined whether there was a reverse causal relationship between urinary stones and circulating lipids (TG, LDL-C, HDL-C, APOA, APOB, and Pure hypercholesterolaemia) along with lipid-lowering drugs (HMGCR inhibitors and PCSK9 inhibitors). We used urinary stones as an exposure factor, circulating lipids and lipid-lowering drugs as outcome factors(We did not extract SNPs associated with exposure factors in the GWAS for HMGCR inhibitors and PCSK9 inhibitors). As anticipated, our reverse Mendelian randomization analysis confirmed the absence of a reverse causal relationship between the genetically predicted circulating lipids, lipid-lowering drugs, and urinary stones (**Supplementary Table 9, Tables S9-S14**).

## 4 Discussions

The primary objective of our study was to investigate the causal relationship between circulating lipid levels and lipid-lowering drugs and their potential impact on the incidence of urinary stones through Mendelian Randomization analysis. Following the rigorous application of MR techniques, we identified a causal link between elevated serum TG levels and an increased risk of urinary stones. In contrast, elevated serum levels of LDL-C, HDL-C, APOA, APOB, Pure hypercholesterolaemia, as well as the use of lipid-lowering drugs such as HMGCR inhibitors and PCSK9 inhibitors, did not exhibit statistically significant associations with either an increased or decreased risk of urinary stones. In other words, no causal relationship was established between these lipid parameters and the development of urinary stones. Importantly, our analysis did not detect significant directional pleiotropy, further bolstering the robustness and reliability of our results. These findings contribute to our understanding of the complex interplay between lipid metabolism and urinary stone formation, emphasizing that while elevated serum TG levels may pose a risk, other lipid-related factors and lipid-lowering medications do not appear to be major contributors to the development of urinary stones. It is important to note that our study is in line with the principles of Mendelian randomization, leveraging genetic variants as instrumental variables to infer causality. However, it is essential to consider the limitations of our study, including the potential for residual confounding and the generalizability of our findings to diverse populations. Future research should delve deeper into the mechanistic links between lipid metabolism and urinary stone formation to provide a more comprehensive understanding of this complex relationship.

In previously published studies, the associations between serum lipids, lipid-lowering drugs, and urinary stones have yielded results that are not entirely consistent and, in some cases, even contradictory. For instance, in a comprehensive population-based follow-up analysis, it was observed that individuals with an elevated plasma cholesterol/HDL-C ratio faced a 1.381-fold increased risk of kidney stone disease (KSD). Paradoxically, low levels of HDL-C were found to provide protection against the development of incidental KSD. This intriguing finding underscores the complexity of the relationship between lipid profiles and urinary stone formation (42). In this study conducted by Qi Ding within the Chinese population, it was similarly observed that while TG levels exhibited a notable increase in patients with kidney stones compared to those without, a significant majority of stone-afflicted individuals displayed markedly reduced levels of total cholesterol (TC) and LDL-C (42). Furthermore, there remains inconsistency in the evidence regarding the role of dyslipidemia in pediatric kidney stones. An observational study conducted in Poland, focusing on urinary stone formation in children and adolescents aged 3 to 18 years, found that dyslipidemia was present in 33% of patients with urinary stones. However, the data also indicated that a high level of LDL-C appeared to play a significant role in hypobarbituria, a condition linked to renal stone formation. These varying observations emphasize the need for further research and exploration to unravel the intricate relationship between lipid profiles and urinary stone risk (43).

Our findings, which reveal a link between triglyceride abnormalities and urinary stones, align with a body of evidence derived from recent cross-sectional analyses and case-control studies (44–50). One noteworthy study, conducted by Ho Won and spanning 7 years with propensity score matching, demonstrated that individuals with urinary stones were more likely to present with hypertriglyceridemia and hypo-HDL-cholesterolemia. Importantly, hypertriglyceridemia emerged as an independent risk factor associated with an increased likelihood of stone recurrence in patients with urolithiasis (51). Furthermore, a prospective cohort study with a 7-year follow-up period unveiled that hypertriglyceridemia heightened the risk of KSD (52). Likewise, two retrospective cohort studies, encompassing over 5 years of follow-up data, disclosed that individuals with dyslipidemia faced a higher risk of developing KSD as well as experiencing recurrent KSD (53, 54). Intriguingly, Feng (55), in an investigation that adjusted for independent risk factors such as gender and age in the context of kidney stone development, screened 11,827 patients for the co-occurrence of metabolic syndrome and kidney stones. The study revealed a noteworthy trend: an increased incidence of kidney stones was associated with rising blood TG levels and declining levels of lipoprotein cholesterol (P<0.05), underscoring a typical positive correlation. Inci et al., in a similar study conducted in a Turkish population, obtained results consistent with other studies (56).

Examining the causal mechanisms related to the components of urinary stones and urinary metabolism, high triglyceride levels also elevate the urinary excretion of components such as calcium, sodium, and potassium. This phenomenon significantly increases the supersaturation of uric acid and creatinine, thereby expediting the formation of urinary stones (57). The presence of lipids in urinary stones was confirmed through histochemical and biochemical methods (58), particularly in the composition of calcium oxalate stones, among others, affirming the close association between urinary stones and lipids (59). Similar urinary findings in patients with dyslipidemia have been reported in other studies (45, 54).

When examining the association between lipid-lowering drugs and urologic diseases, certain researchers (60, 61) have reported a noteworthy observation. They found that individuals who initiated statin therapy exhibited a significantly reduced likelihood of developing new urinary stones in comparison to those who did not receive statin treatment. This protective effect was notably more pronounced among individuals with a history of prior stone formation. However, it is essential to emphasize that the underlying mechanism driving this effect appears to diverge from the conventional lipid-lowering properties of statins. In a study conducted by Liu et al. (62), it was elucidated that statin therapy may potentially mitigate the risk of uric acid stone formation by inducing alterations in the urinary composition of patients with KSD. This included an increase in urinary pH levels and citrate concentration. Despite these promising findings and the potential clinical benefits of statin therapy for individuals with KSD, our own investigations did not uncover a causal relationship between statin use and the risk of urinary stones. It is important to underscore that while statins may offer protective effects against stone formation, our Mendelian randomization analysis did not reveal a direct causal link between statin therapy and urinary stones. Further research is needed to elucidate the precise mechanisms underpinning these observations and to consolidate our understanding of the relationship between statins and urologic diseases.

Our study leveraged a robust dataset comprising recent urological stone cohort studies with large sample sizes. Notably, the participants from the UK Biobank included in the analysis, encompassing the five selected circulating lipids as exposures, were sourced from the same experimental study. An exception was made for Pure hypercholesterolaemia, which was derived from a distinct cohort. This harmonization of exposure sources offered a notable advantage, eliminating the potential error associated with using different sample sources for various exposures. As a result, our study was empowered to detect and establish a causal relationship between circulating lipids and lipid-lowering drugs in relation to the risk of urinary stones, at least within the European population. The robustness of our findings is underscored by the compelling and consistent causal links observed across different exposures and outcomes. Specifically, based on a two-sample MR analysis incorporating multiple exposures, our study provides support for the assertion that elevated serum TG levels are associated with an increased risk of urinary stones. However, we did not observe a significant association between other lipid parameters, as well as lipid-lowering medications, and the risk of urinary stones. While our findings align with some previous research, they offer enhanced credibility by providing genetic-level evidence. Nevertheless, it’s imperative to acknowledge certain limitations in our analysis. Firstly, all our GWAS data rely on European populations, primarily from the UK Biobank. This geographical focus could introduce bias stemming from differences in ethnicity, environmental factors, and dietary habits (63–66). Secondly, despite identifying a significant causal relationship between TG levels and urinary stone occurrence through available MR analyses, we cannot definitively exclude the possibility of key mediators bridging elevated TG and the occurrence of urinary stones due to inherent methodological constraints. Consequently, further investigations are warranted to delve deeper into the intricate relationship between lipid profiles and urinary stones, thereby validating our current findings.

## 5 Conclusion

Our findings suggest that elevated triglyceride levels may heighten the risk of urinary stones. Consequently, individuals with urolithiasis should consider enhanced monitoring of triglyceride metabolism. However, this Mendelian randomization study did not reveal any significant associations between LDL-C, HDL-C, APOA, APOB, pure hypercholesterolemia, or lipid-lowering drugs and urinary stones.

## Data availability statement

The original contributions presented in the study are included in the article/**Supplementary Material**. Further inquiries can be directed to the corresponding author.

## Ethics statement

This study only used published or publicly available data. Ethical approval for each study included in the investigation can be found in the original publications (including informed consent from each participant). The studies were conducted in accordance with the local legislation and institutional requirements. The participants provided their written informed consent to participate in this study.

## Author contributions

ZT: Data curation, Writing – original draft, Writing – review & editing, Methodology. JH: Data curation, Writing – review & editing. AS: Data curation, Visualization, Writing – review & editing. MD: Methodology, Project administration, Writing – review & editing. JS: Writing – review & editing, Data curation, Funding acquisition.
