# Supplementary material for: Causal effects of circulating lipids and lipid-lowering drugs on the risk of urinary stones: a Mendelian randomization study
Source: Front Endocrinol (Lausanne). 2023 Dec 1;14:1301163. doi: 10.3389/fendo.2023.1301163 (PMC10722409; doi:10.3389/fendo.2023.1301163)
Supplement: Supplementary file 1 [file DataSheet_1.zip › Data Sheet 1.PDF]

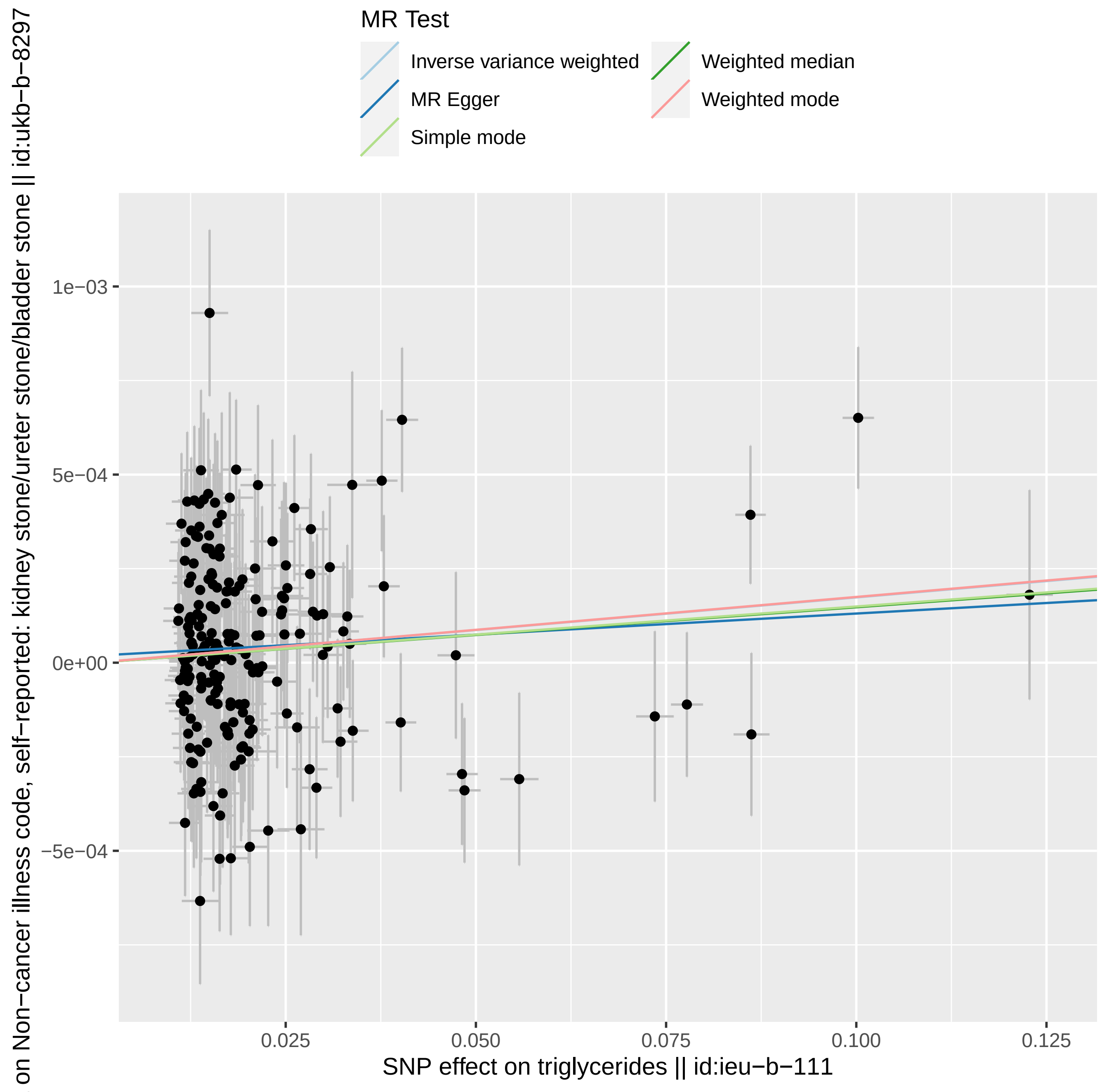

MR Test
Inverse variance weighted
MR Egger
Simple mode
Weighted median
Weighted mode
1e−03
5e−04
0e+00
−5e−04
0.025
0.050
0.075
0.100
0.125
SNP effect on triglycerides || id:ieu−b−111
on Non−cancer illness code, self−reported: kidney stone/ureter stone/bladder stone || id:ukb−b−8297

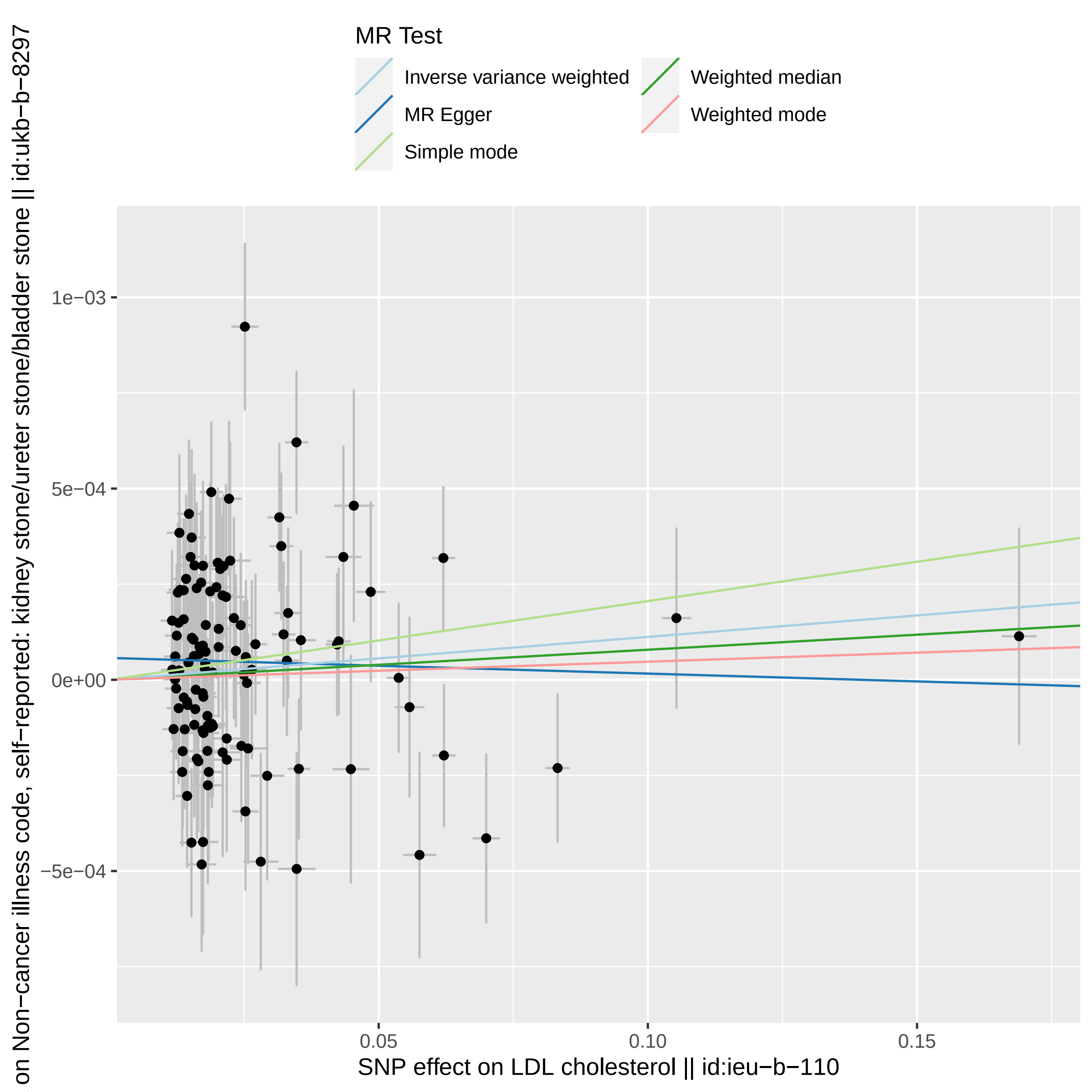
MR Test
Inverse variance weighted
MR Egger
Simple mode
Weighted median
Weighted mode
1e−03
5e−04
0e+00
−5e−04
0.05
0.10
0.15
SNP effect on LDL cholesterol || id:ieu−b−110
on Non−cancer illness code, self−reported: kidney stone/ureter stone/bladder stone || id:ukb−b−8297

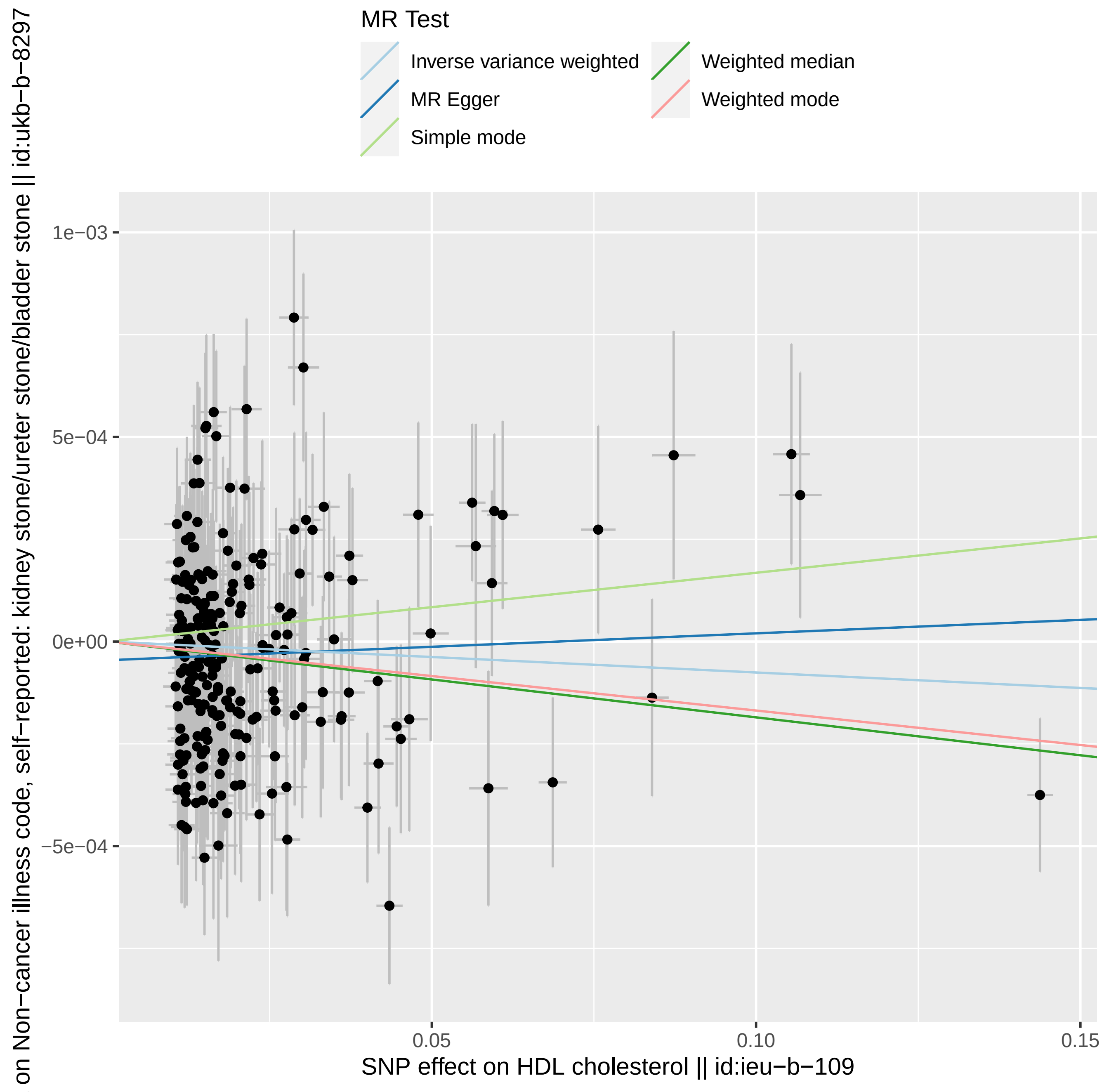
MR Test
Inverse variance weighted
MR Egger
Simple mode
Weighted median
Weighted mode
1e−03
5e−04
0e+00
−5e−04
0.05
0.10
0.15
SNP effect on HDL cholesterol || id:ieu−b−109
on Non−cancer illness code, self−reported: kidney stone/ureter stone/bladder stone || id:ukb−b−8297

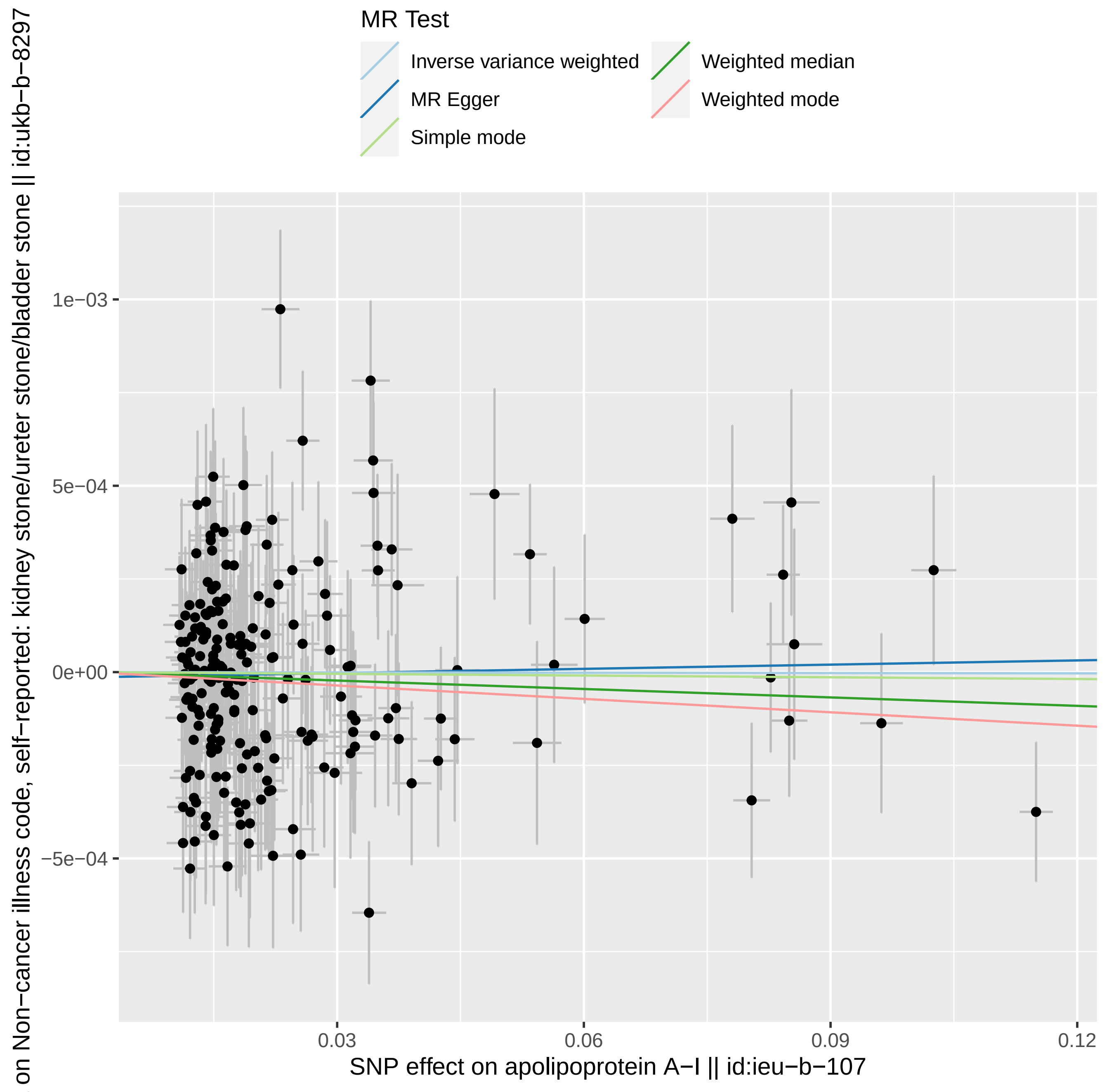

MR Test
Inverse variance weighted
MR Egger
Simple mode
Weighted median
Weighted mode
1e−03
5e−04
0e+00
−5e−04
0.03
0.06
0.09
0.12
SNP effect on apolipoprotein A−I || id:ieu−b−107
on Non−cancer illness code, self−reported: kidney stone/ureter stone/bladder stone || id:ukb−b−8297

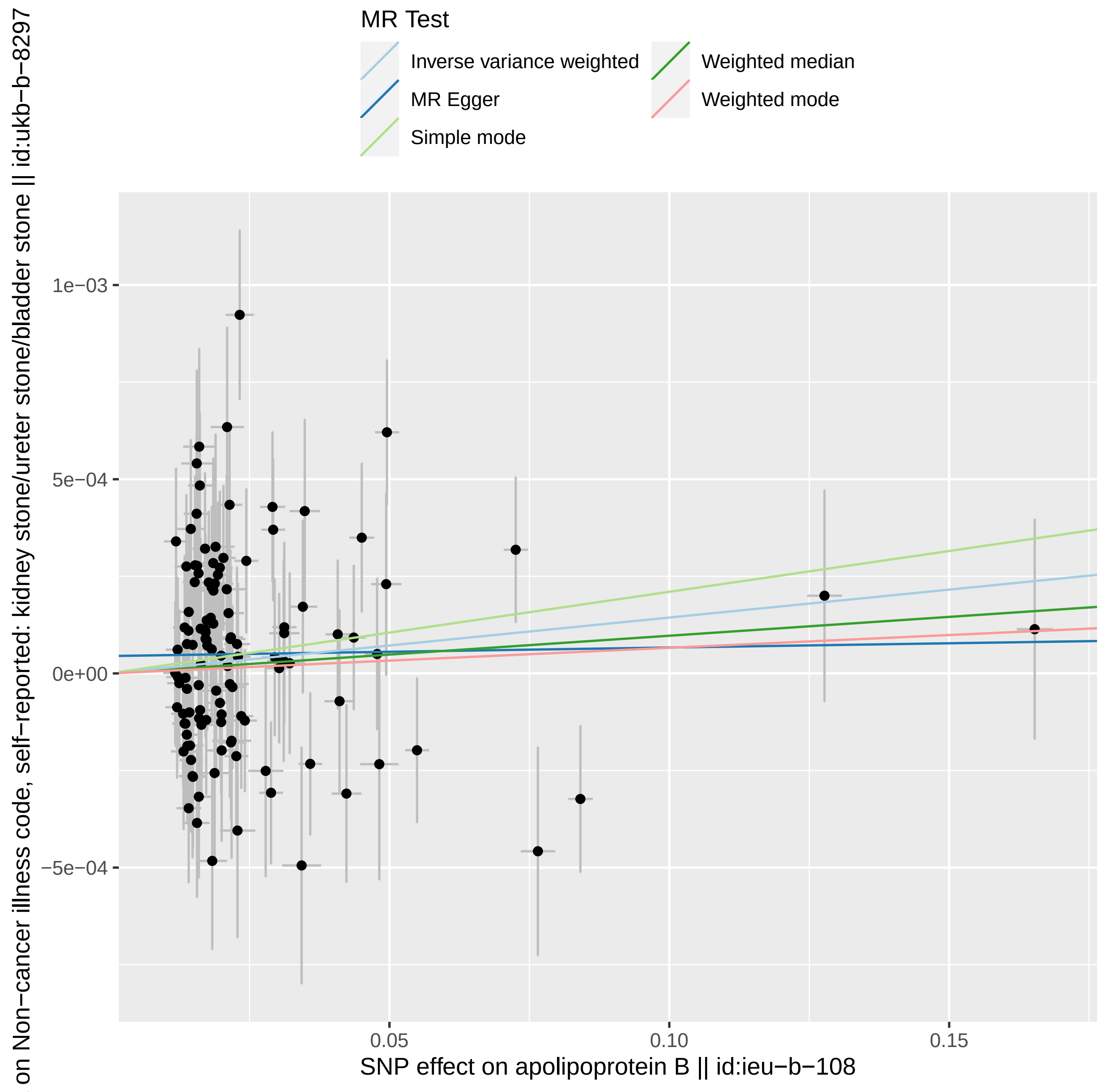

MR Test
Inverse variance weighted
MR Egger
Simple mode
Weighted median
Weighted mode
1e−03
5e−04
0e+00
−5e−04
0.05
0.10
0.15
SNP effect on apolipoprotein B || id:ieu−b−108
on Non−cancer illness code, self−reported: kidney stone/ureter stone/bladder stone || id:ukb−b−8297

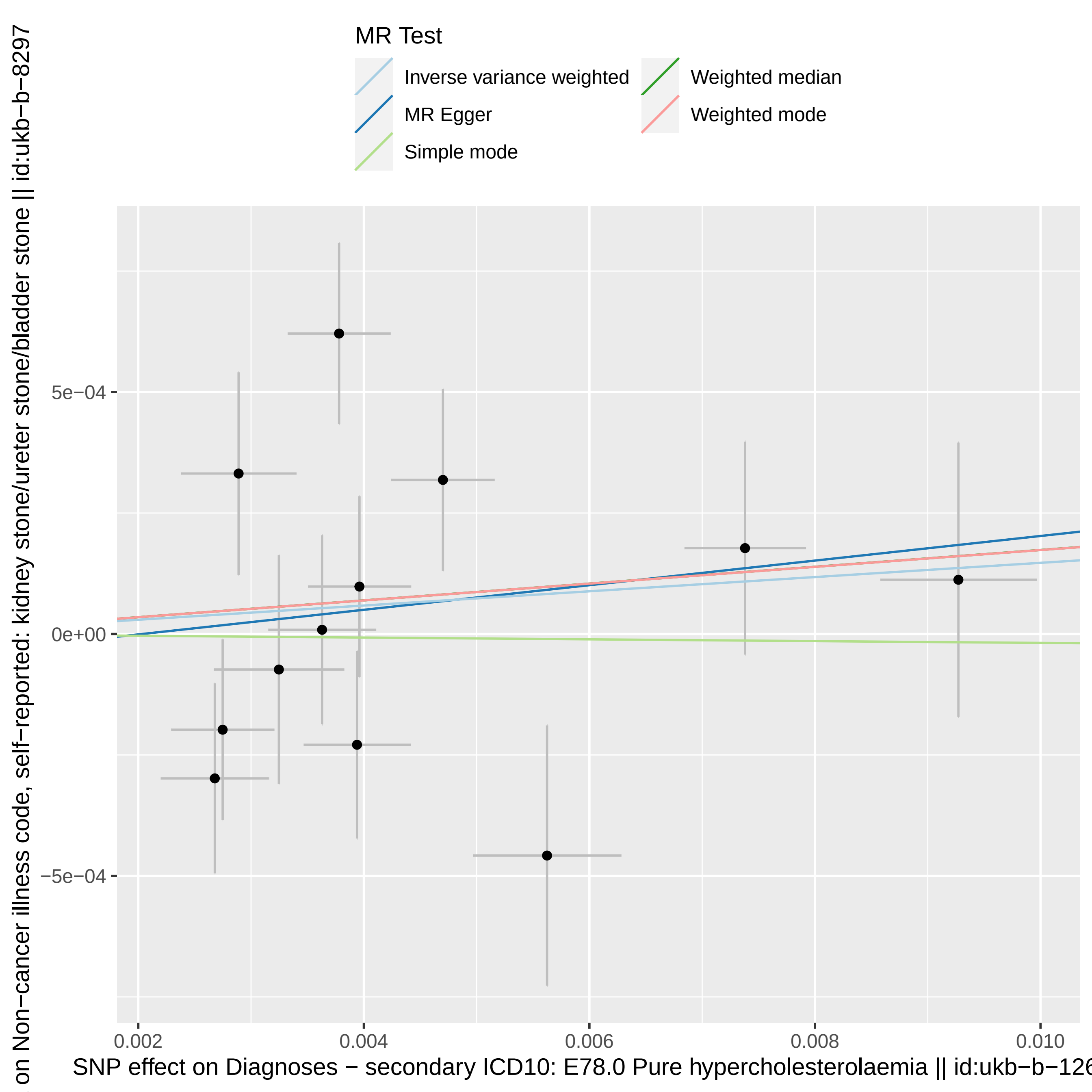

MR Test
Inverse variance weighted
MR Egger
Simple mode
Weighted median
Weighted mode
5e−04
0e+00
−5e−04
0.002
0.004
0.006
0.008
0.010
SNP effect on Diagnoses − secondary ICD10: E78.0 Pure hypercholesterolaemia || id:ukb−b−126
on Non−cancer illness code, self−reported: kidney stone/ureter stone/bladder stone || id:ukb−b−8297

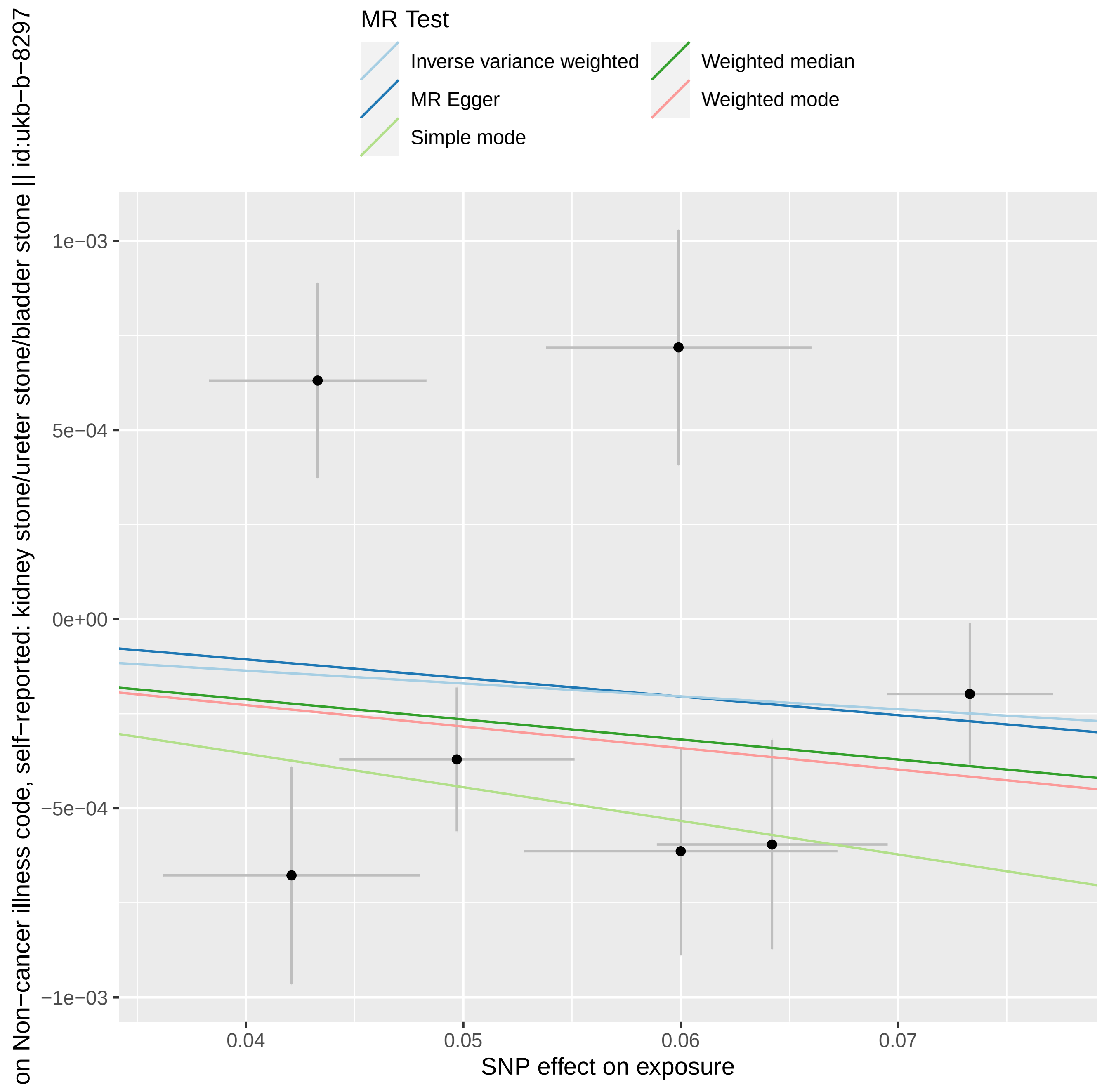

MR Test
Inverse variance weighted
MR Egger
Simple mode
Weighted median
Weighted mode
1e−03
5e−04
0e+00
−5e−04
−1e−03
0.04
0.05
0.06
0.07
SNP effect on exposure
on Non−cancer illness code, self−reported: kidney stone/ureter stone/bladder stone || id:ukb−b−8297

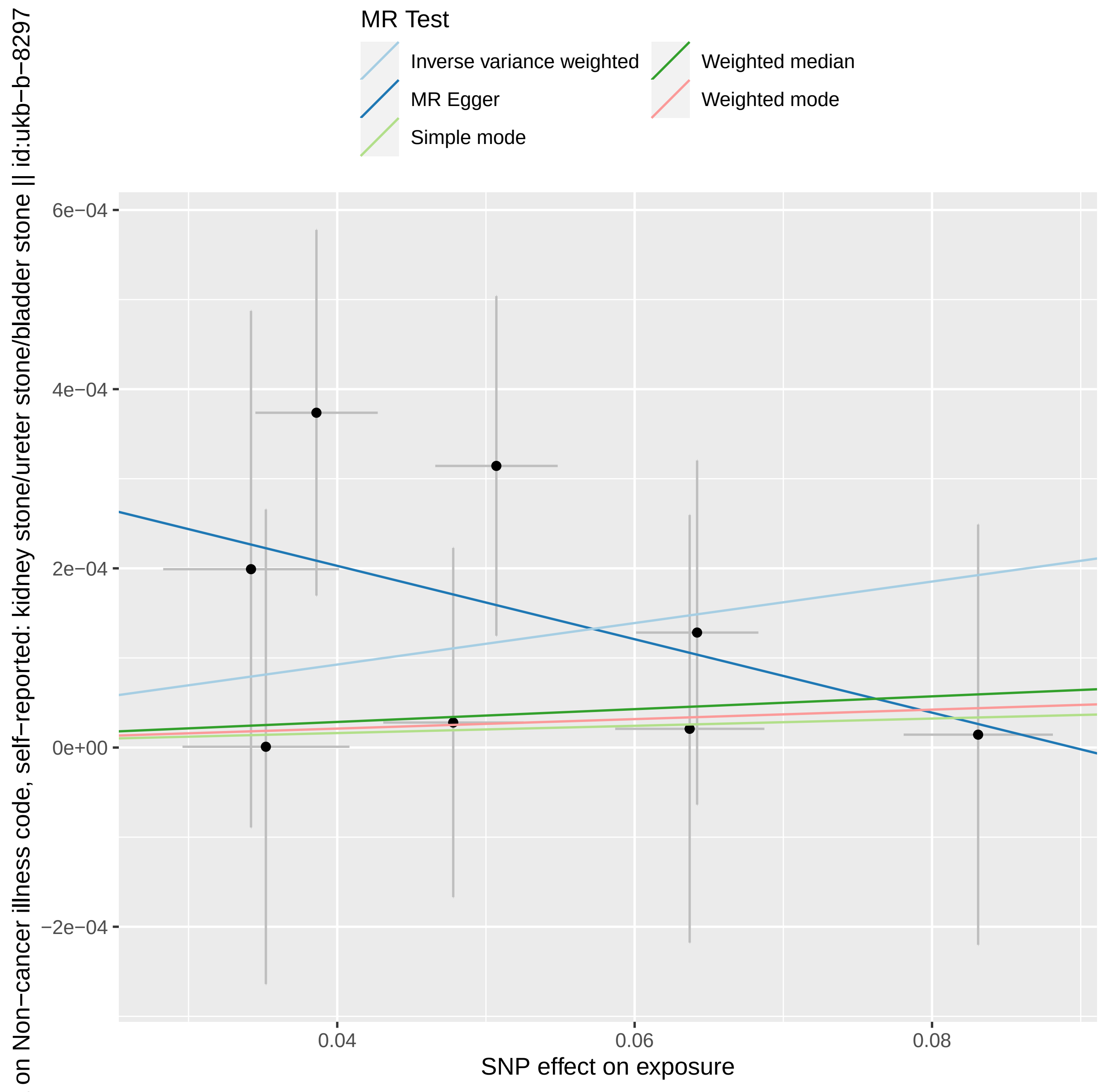
MR Test
Inverse variance weighted
MR Egger
Simple mode
Weighted median
Weighted mode
6e−04
4e−04
2e−04
0e+00
−2e−04
0.04
0.06
0.08
SNP effect on exposure
on Non−cancer illness code, self−reported: kidney stone/ureter stone/bladder stone || id:ukb−b−8297
