# Supplementary material for: Causal effects of circulating lipids and lipid-lowering drugs on the risk of urinary stones: a Mendelian randomization study
Source: Front Endocrinol (Lausanne). 2023 Dec 1;14:1301163. doi: 10.3389/fendo.2023.1301163 (PMC10722409; doi:10.3389/fendo.2023.1301163)
Supplement: Supplementary file 1 [file DataSheet_1.zip › Data Sheet 2.PDF]

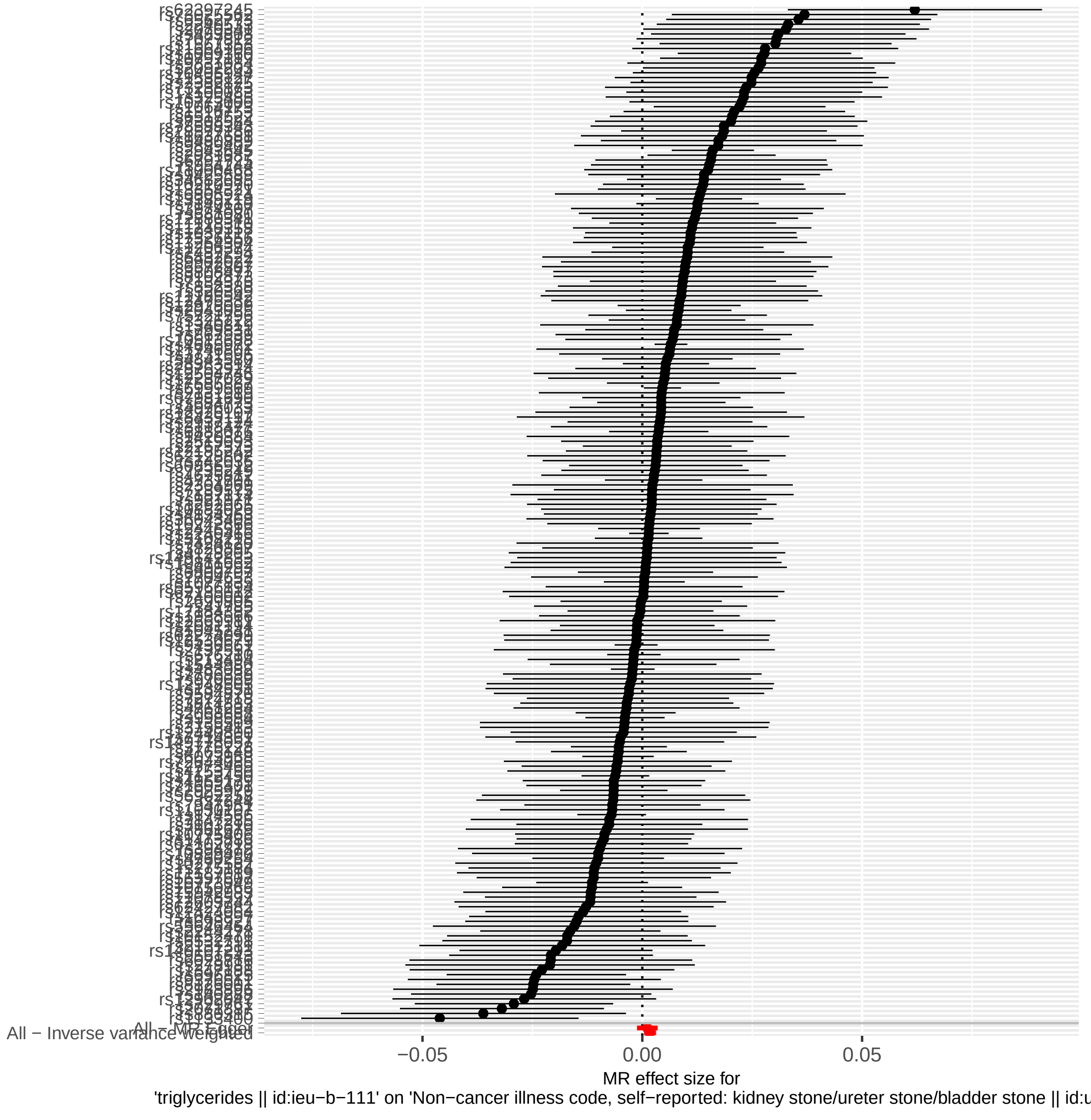

rs62397245
All − MR Egger
All − Inverse variance weighted
−0.05
0.00
0.05
MR effect size for
'triglycerides || id:ieu−b−111' on 'Non−cancer illness code, self−reported: kidney stone/ureter stone/bladder stone || id:u

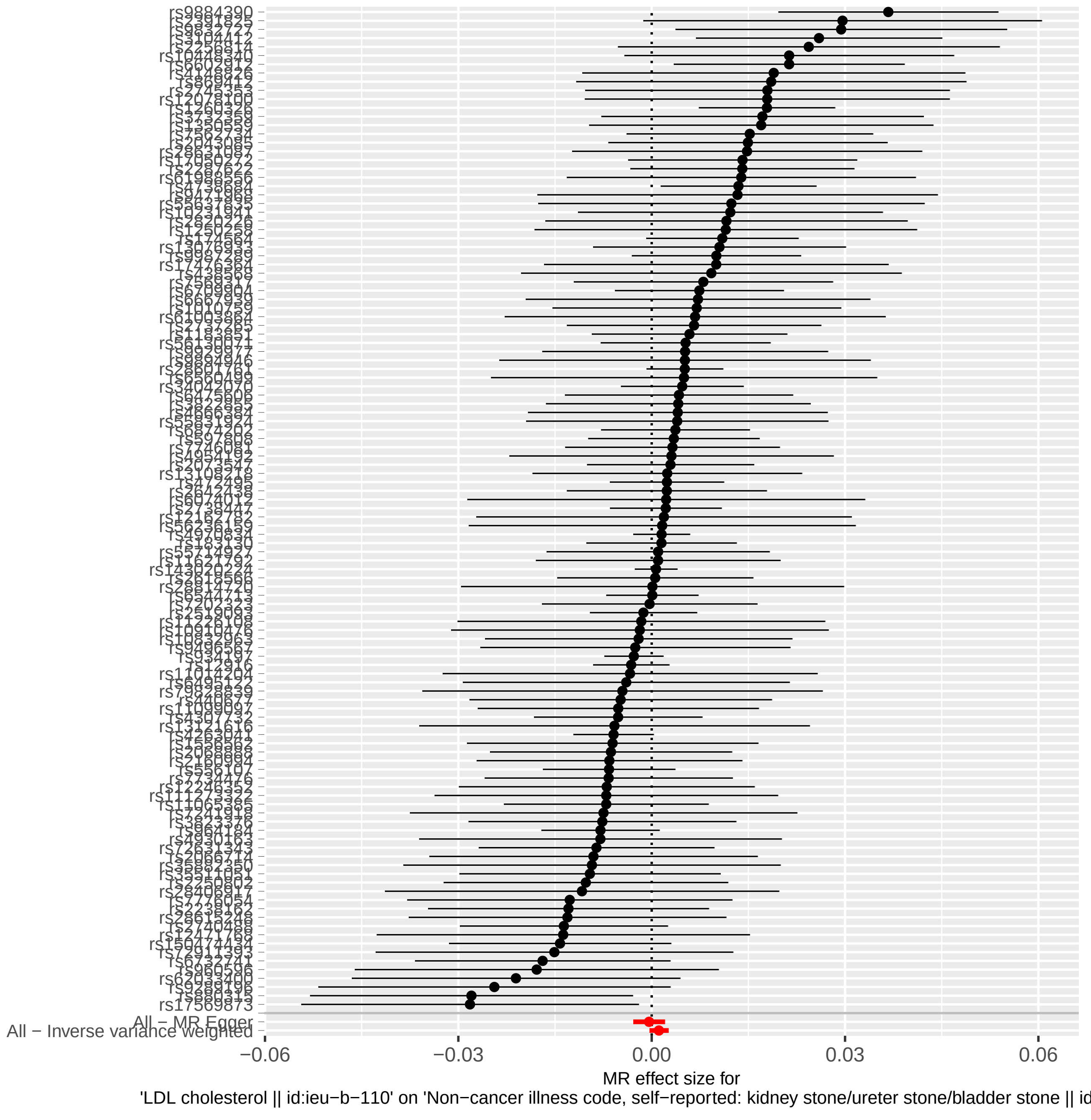
rs9884390
rs17569873
All − MR Egger
All − Inverse variance weighted
−0.06
−0.03
0.00
0.03
0.06
MR effect size for
'LDL cholesterol || id:ieu−b−110' on 'Non−cancer illness code, self−reported: kidney stone/ureter stone/bladder stone || id

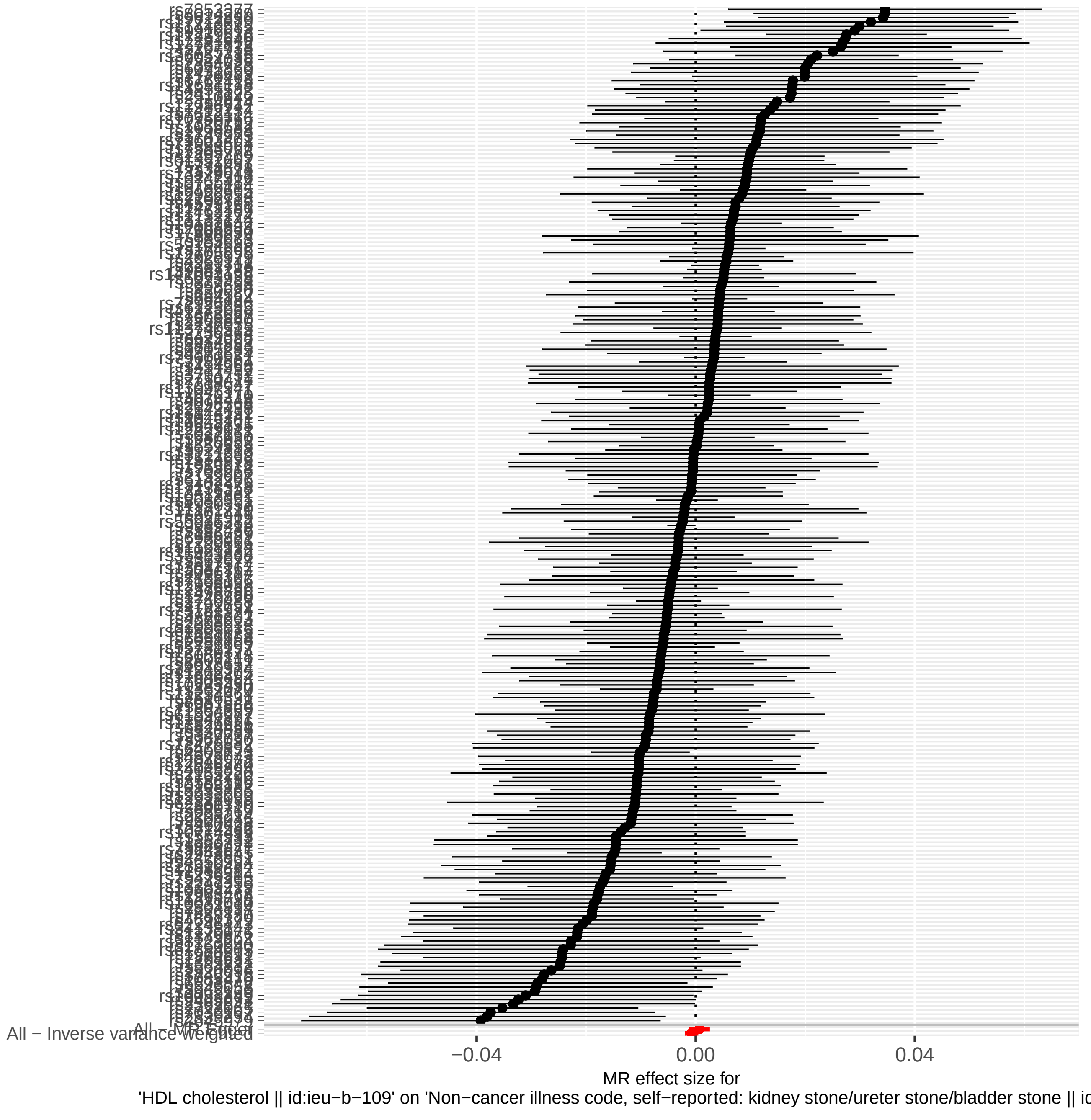
All − MR Egger
All − Inverse variance weighted
−0.04
0.00
0.04
MR effect size for
'HDL cholesterol || id:ieu−b−109' on 'Non−cancer illness code, self−reported: kidney stone/ureter stone/bladder stone || id

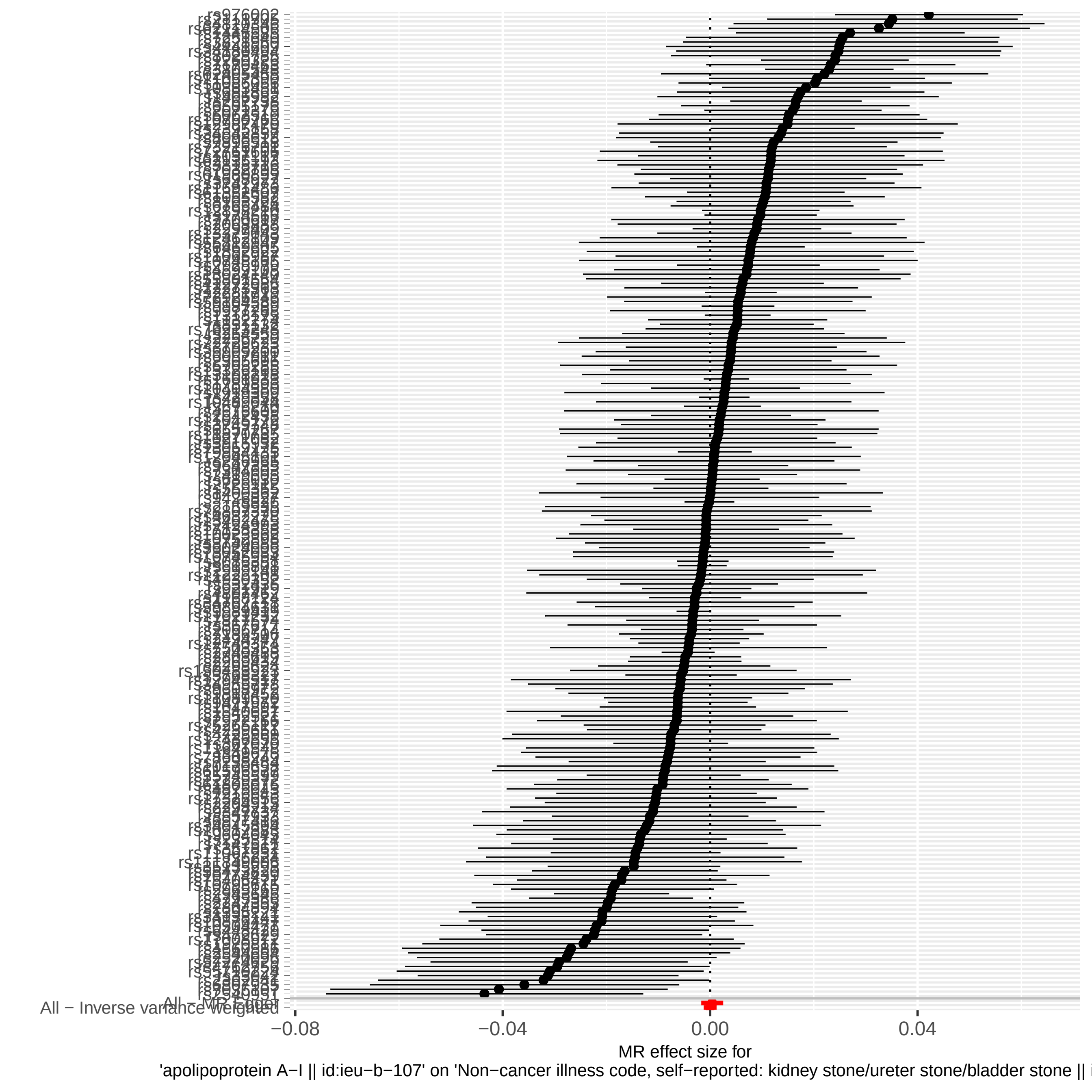

rs976002
rs2940961
All − MR Egger
All − Inverse variance weighted
−0.08
−0.04
0.00
0.04
MR effect size for
'apolipoprotein A−I || id:ieu−b−107' on 'Non−cancer illness code, self−reported: kidney stone/ureter stone/bladder stone || i

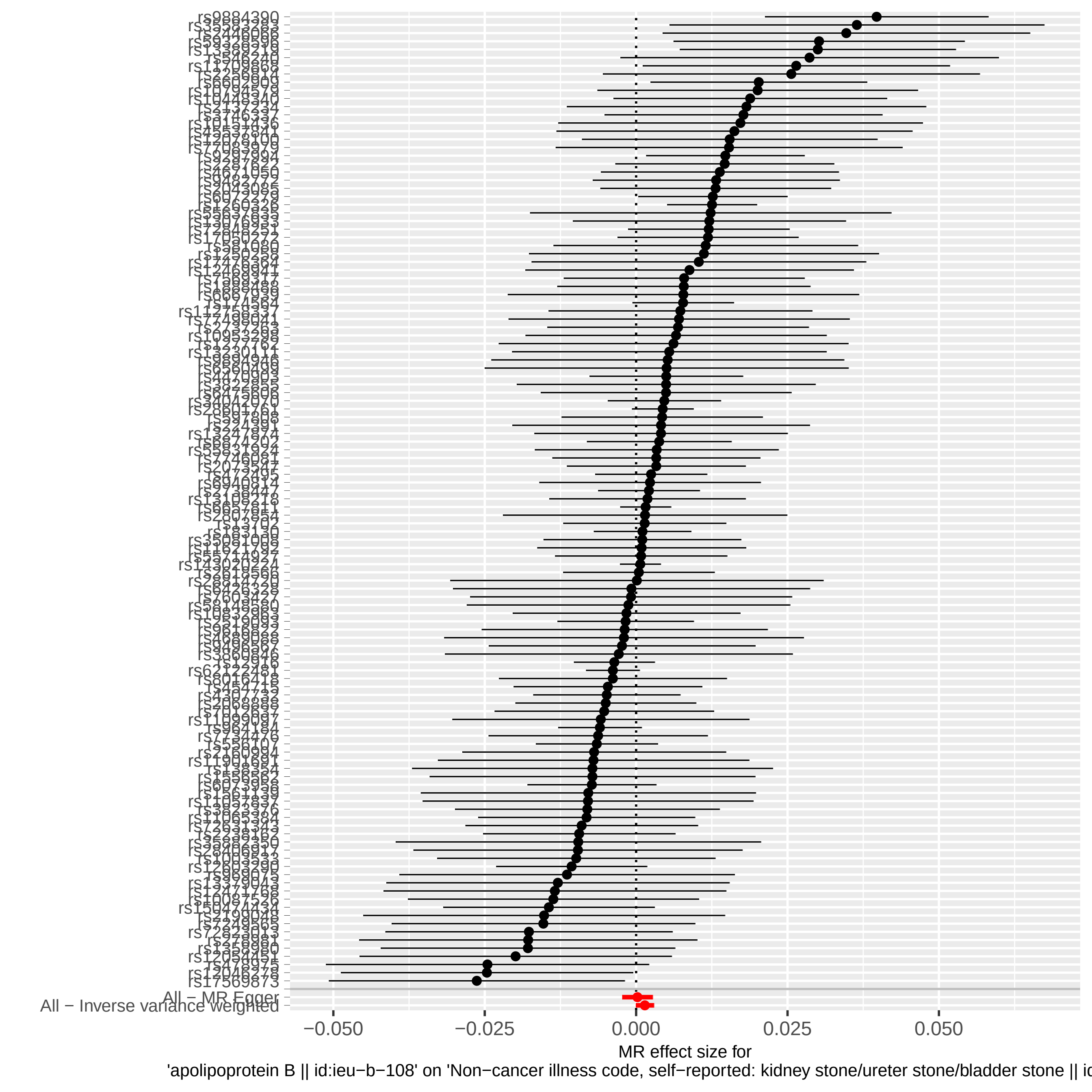
rs9884390
rs17569873
All − MR Egger
All − Inverse variance weighted
−0.050
−0.025
0.000
0.025
0.050
MR effect size for
'apolipoprotein B || id:ieu−b−108' on 'Non−cancer illness code, self−reported: kidney stone/ureter stone/bladder stone || id

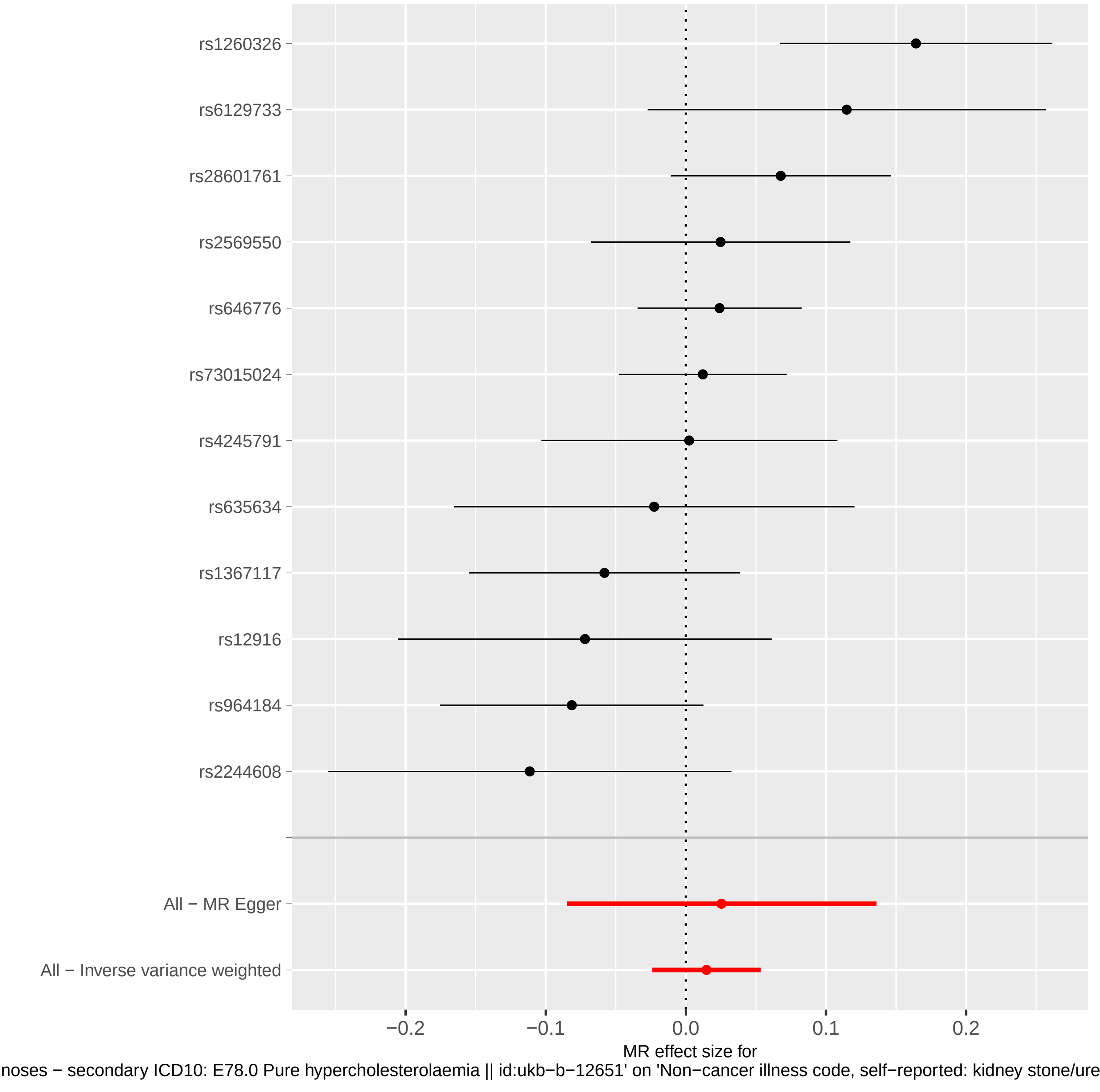
rs1260326
rs6129733
rs28601761
rs2569550
rs646776
rs73015024
rs4245791
rs635634
rs1367117
rs12916
rs964184
rs2244608
All − MR Egger
All − Inverse variance weighted
−0.2
−0.1
0.0
0.1
0.2
MR effect size for
noses − secondary ICD10: E78.0 Pure hypercholesterolaemia || id:ukb−b−12651' on 'Non−cancer illness code, self−reported: kidney stone/ure

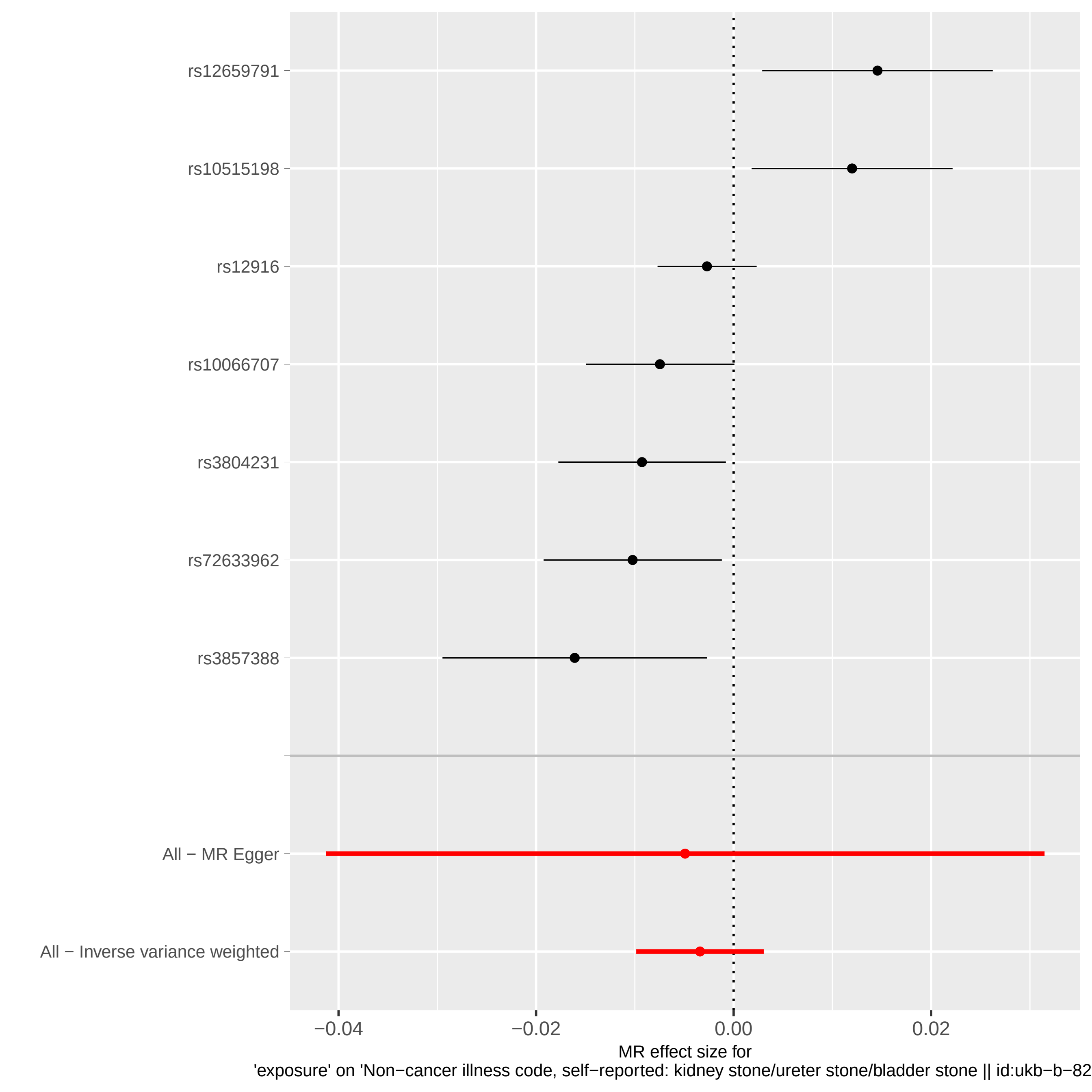
rs12659791
rs10515198
rs12916
rs10066707
rs3804231
rs72633962
rs3857388
All − MR Egger
All − Inverse variance weighted
−0.04
−0.02
0.00
0.02
MR effect size for
'exposure' on 'Non−cancer illness code, self−reported: kidney stone/ureter stone/bladder stone || id:ukb−b−82

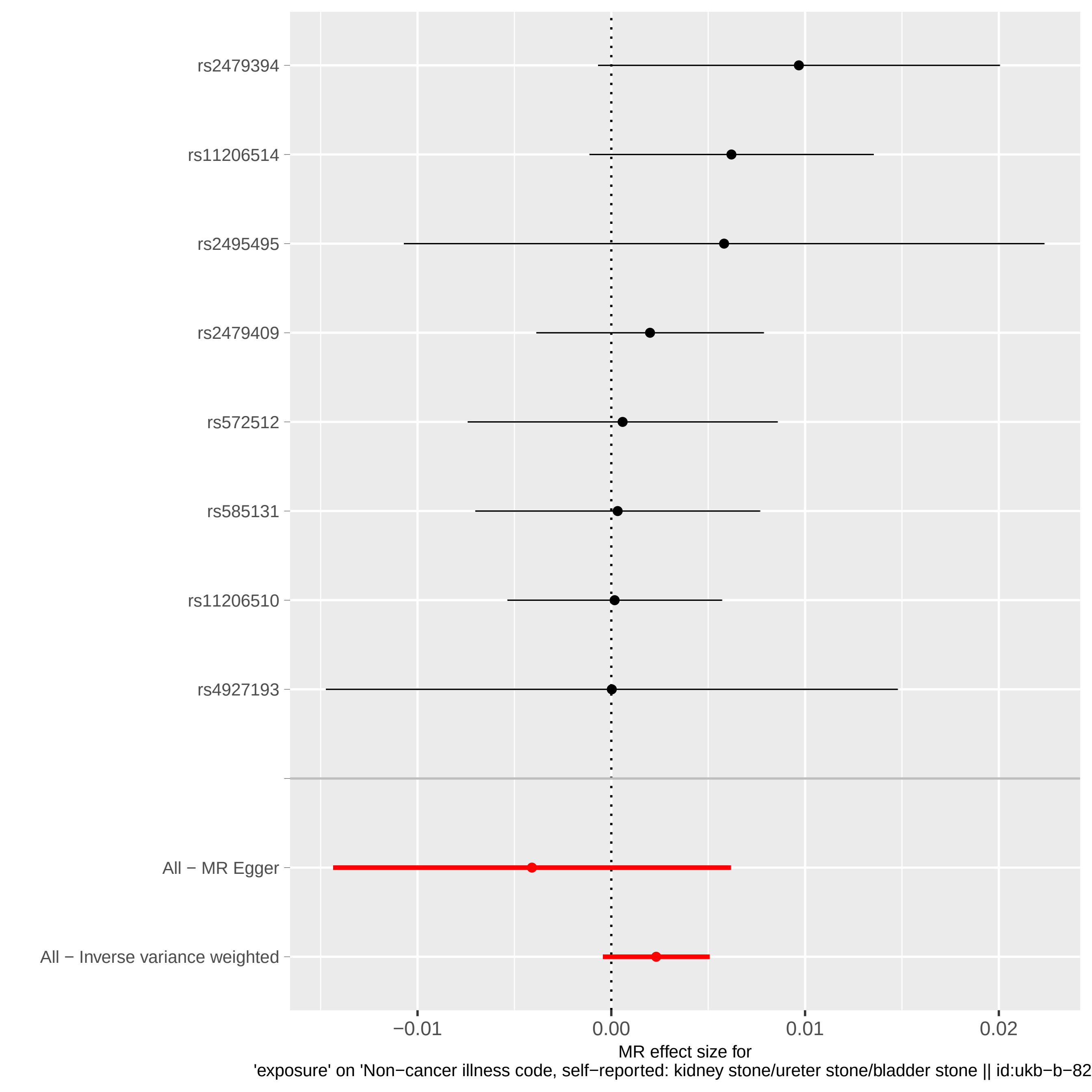
rs2479394
rs11206514
rs2495495
rs2479409
rs572512
rs585131
rs11206510
rs4927193
All − MR Egger
All − Inverse variance weighted
−0.01
0.00
0.01
0.02
MR effect size for
'exposure' on 'Non−cancer illness code, self−reported: kidney stone/ureter stone/bladder stone || id:ukb−b−82
