# Supplementary material for: Causal effects of circulating lipids and lipid-lowering drugs on the risk of urinary stones: a Mendelian randomization study
Source: Front Endocrinol (Lausanne). 2023 Dec 1;14:1301163. doi: 10.3389/fendo.2023.1301163 (PMC10722409; doi:10.3389/fendo.2023.1301163)
Supplement: Supplementary file 1 [file DataSheet_1.zip › Data Sheet 3.PDF]

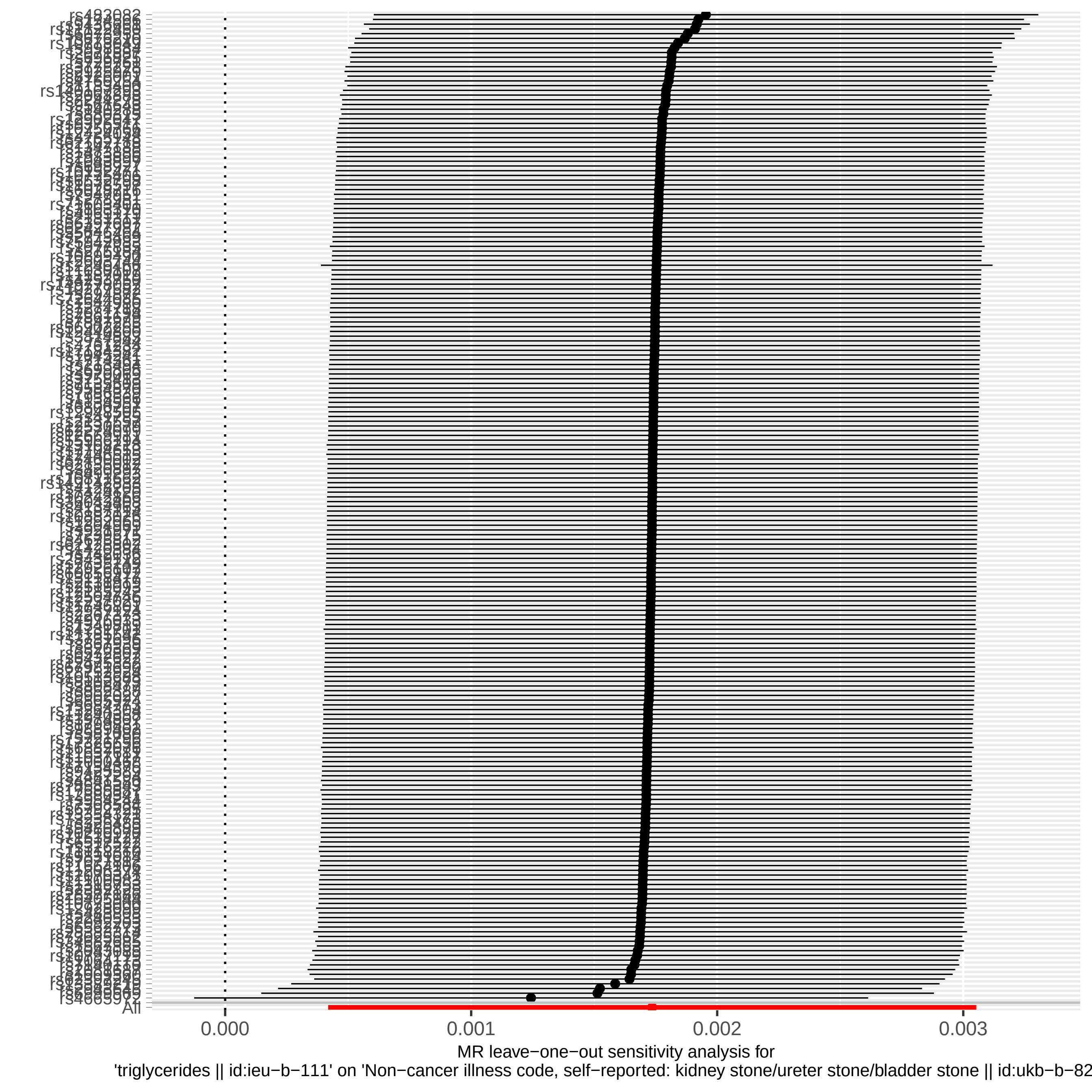
All
0.000
0.001
0.002
0.003
MR leave−one−out sensitivity analysis for
'triglycerides || id:ieu−b−111' on 'Non−cancer illness code, self−reported: kidney stone/ureter stone/bladder stone || id:ukb−b−82

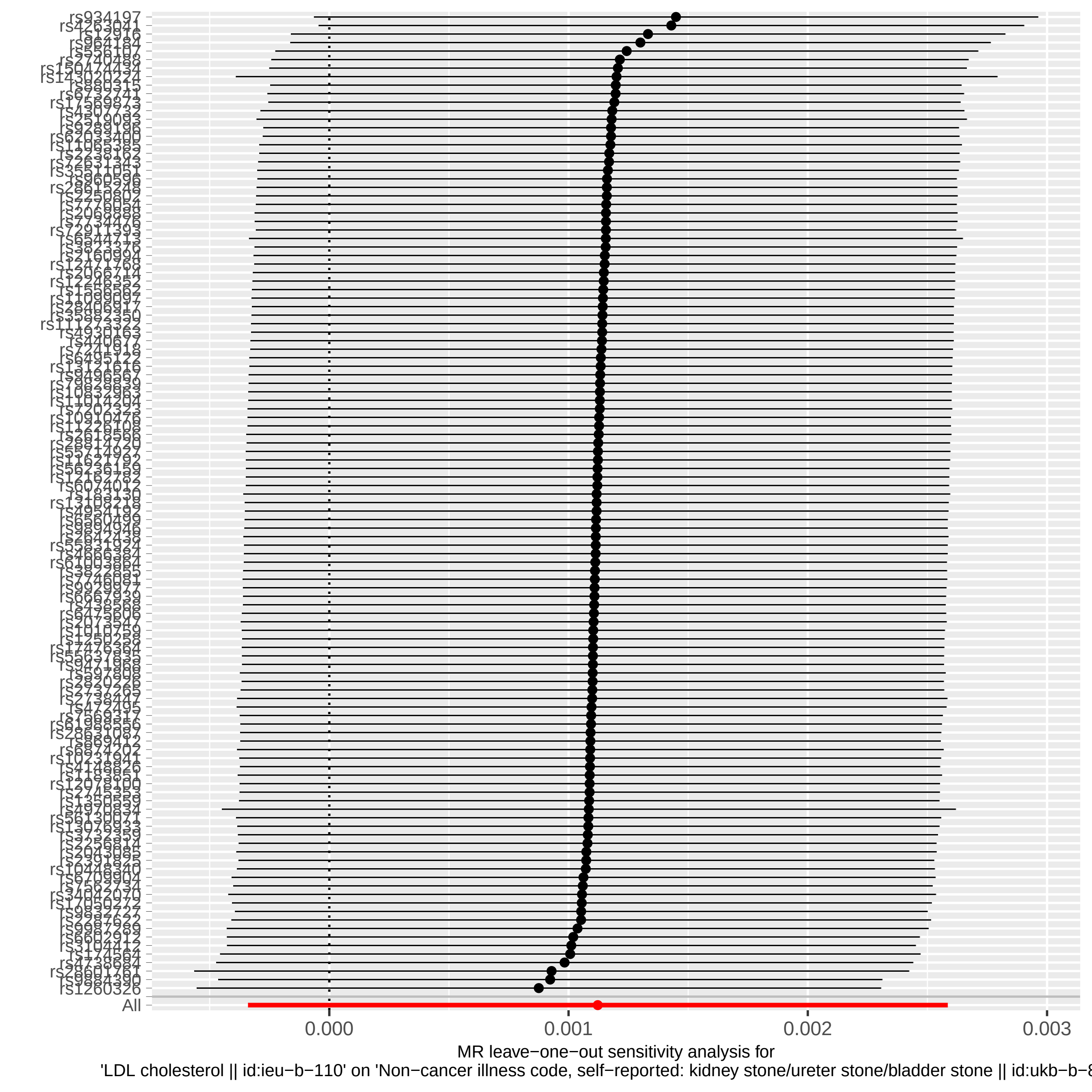
rs934197
rs1260326
All
0.000
0.001
0.002
0.003
MR leave−one−out sensitivity analysis for
'LDL cholesterol || id:ieu−b−110' on 'Non−cancer illness code, self−reported: kidney stone/ureter stone/bladder stone || id:ukb−b−8

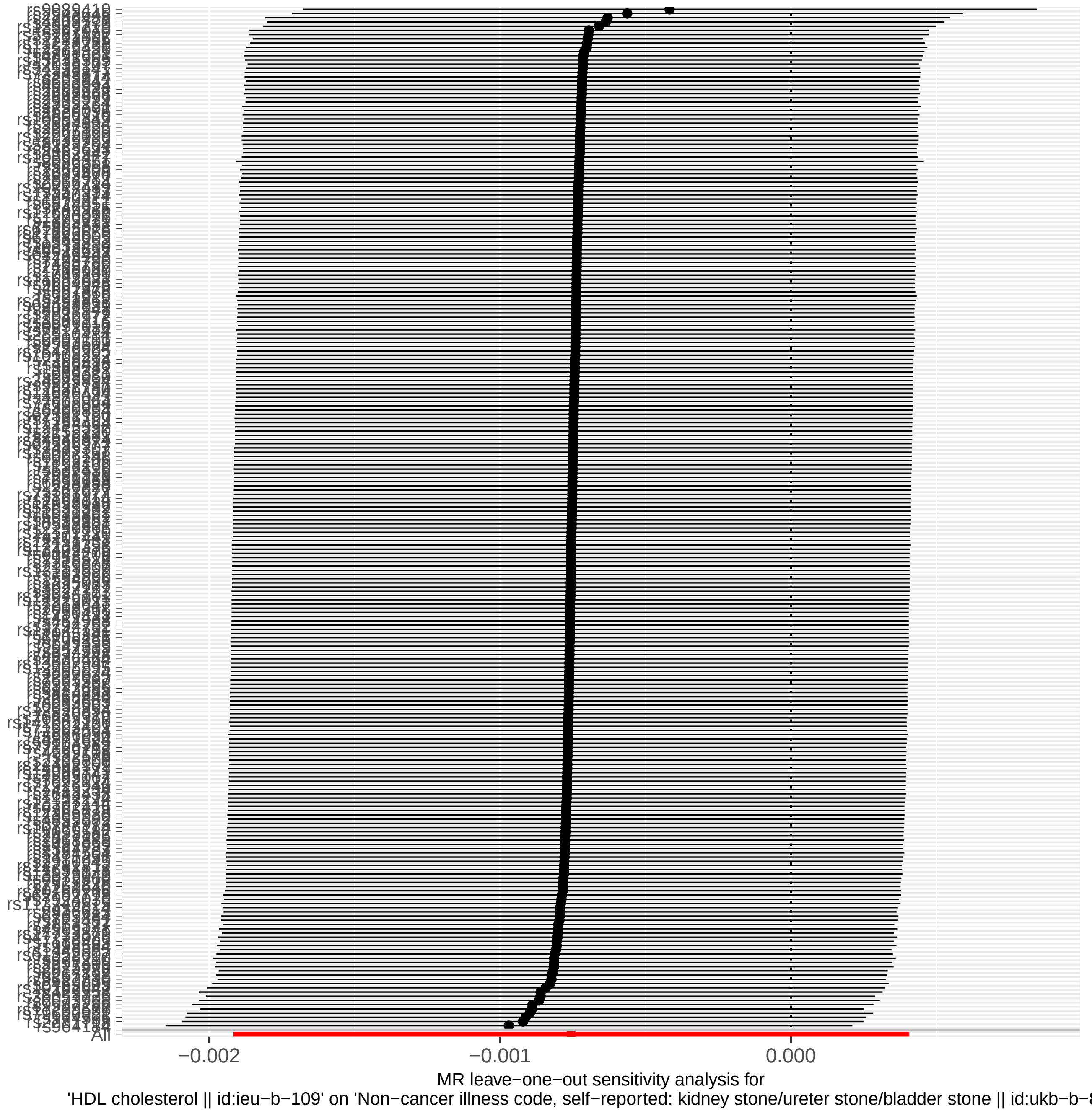
All
−0.002
−0.001
0.000
MR leave−one−out sensitivity analysis for
'HDL cholesterol || id:ieu−b−109' on 'Non−cancer illness code, self−reported: kidney stone/ureter stone/bladder stone || id:ukb−b−8

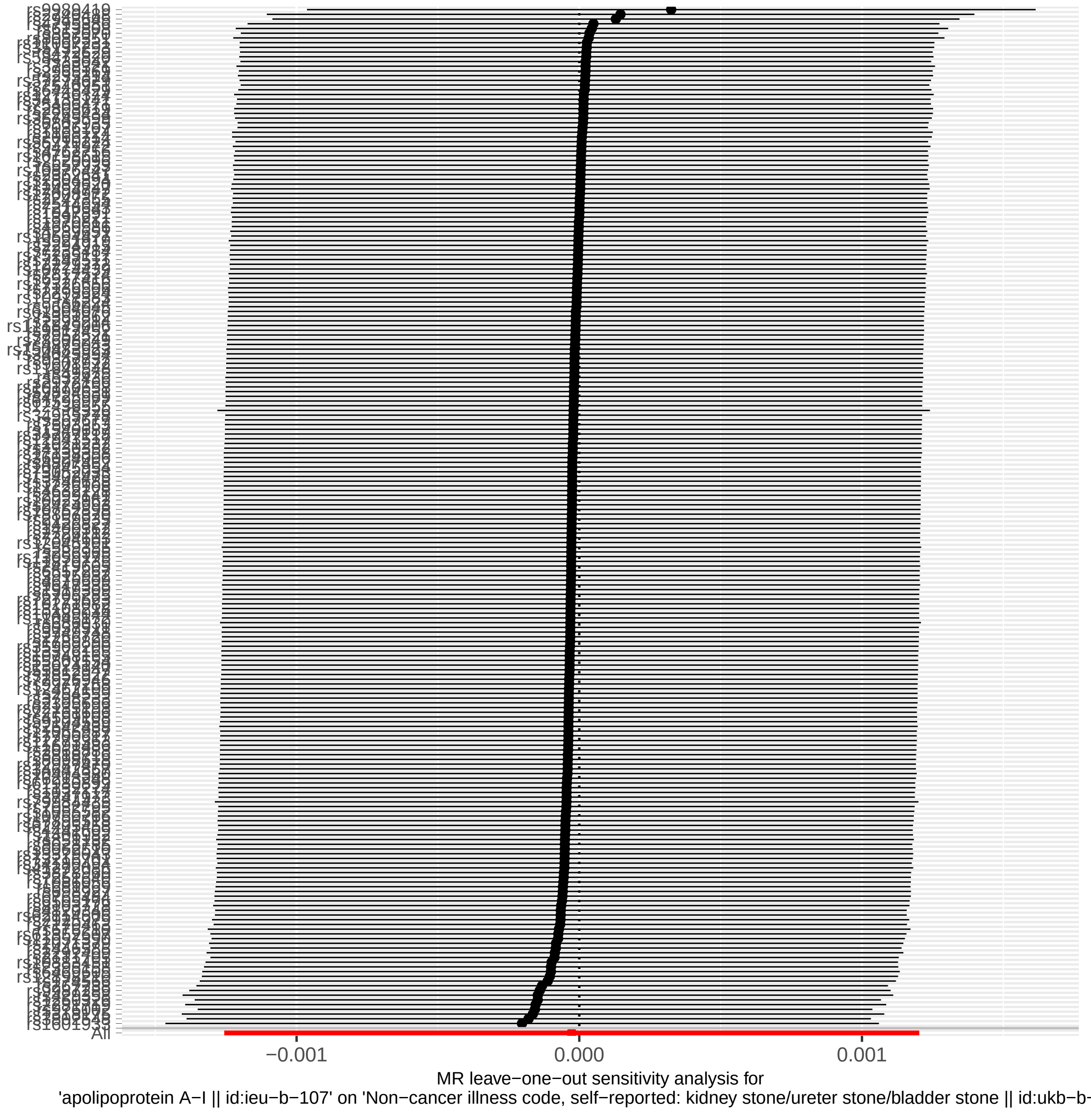
All
−0.001
0.000
0.001
MR leave−one−out sensitivity analysis for
'apolipoprotein A−I || id:ieu−b−107' on 'Non−cancer illness code, self−reported: kidney stone/ureter stone/bladder stone || id:ukb−b−

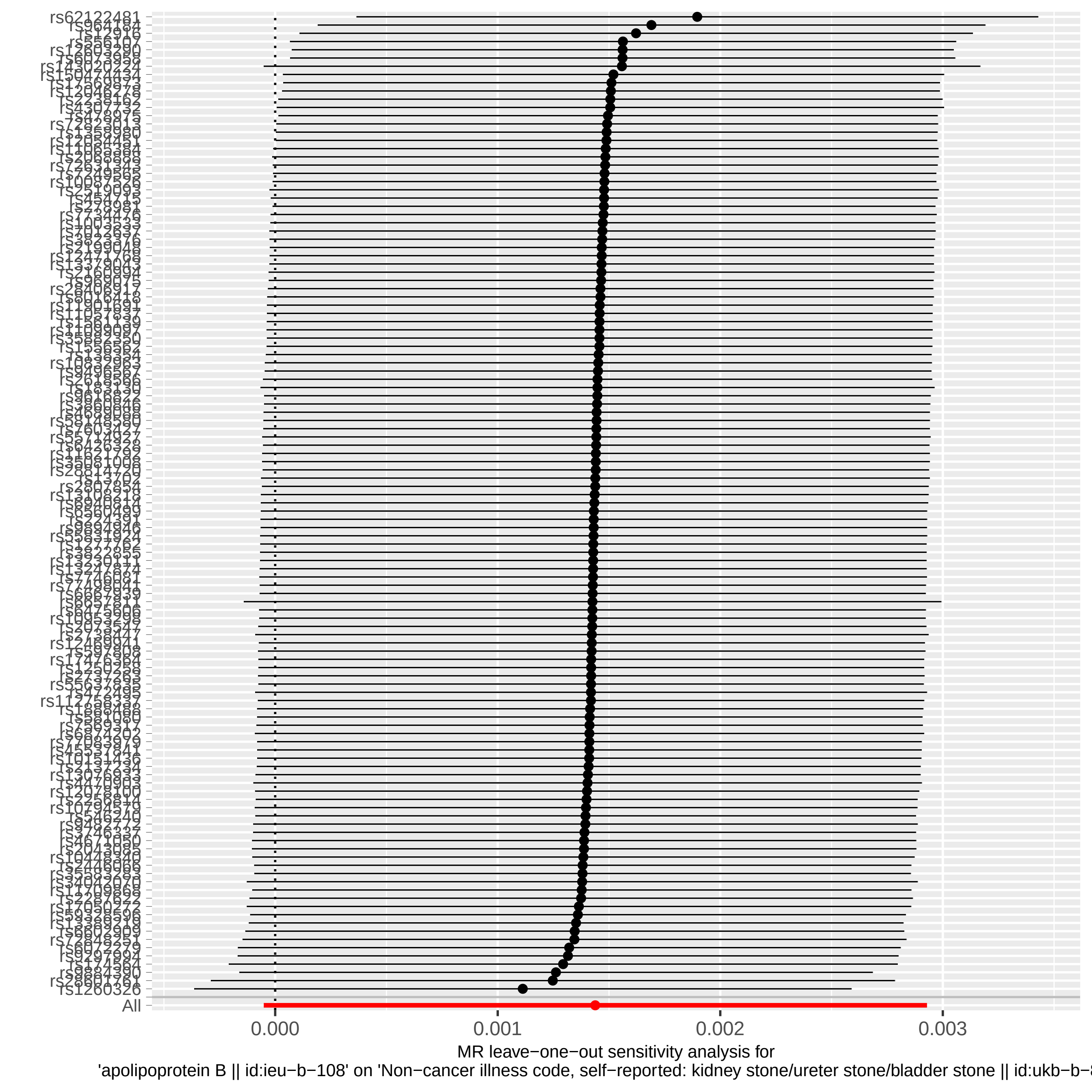
rs62122481
rs1260326
All
0.000
0.001
0.002
0.003
MR leave−one−out sensitivity analysis for
'apolipoprotein B || id:ieu−b−108' on 'Non−cancer illness code, self−reported: kidney stone/ureter stone/bladder stone || id:ukb−b−8

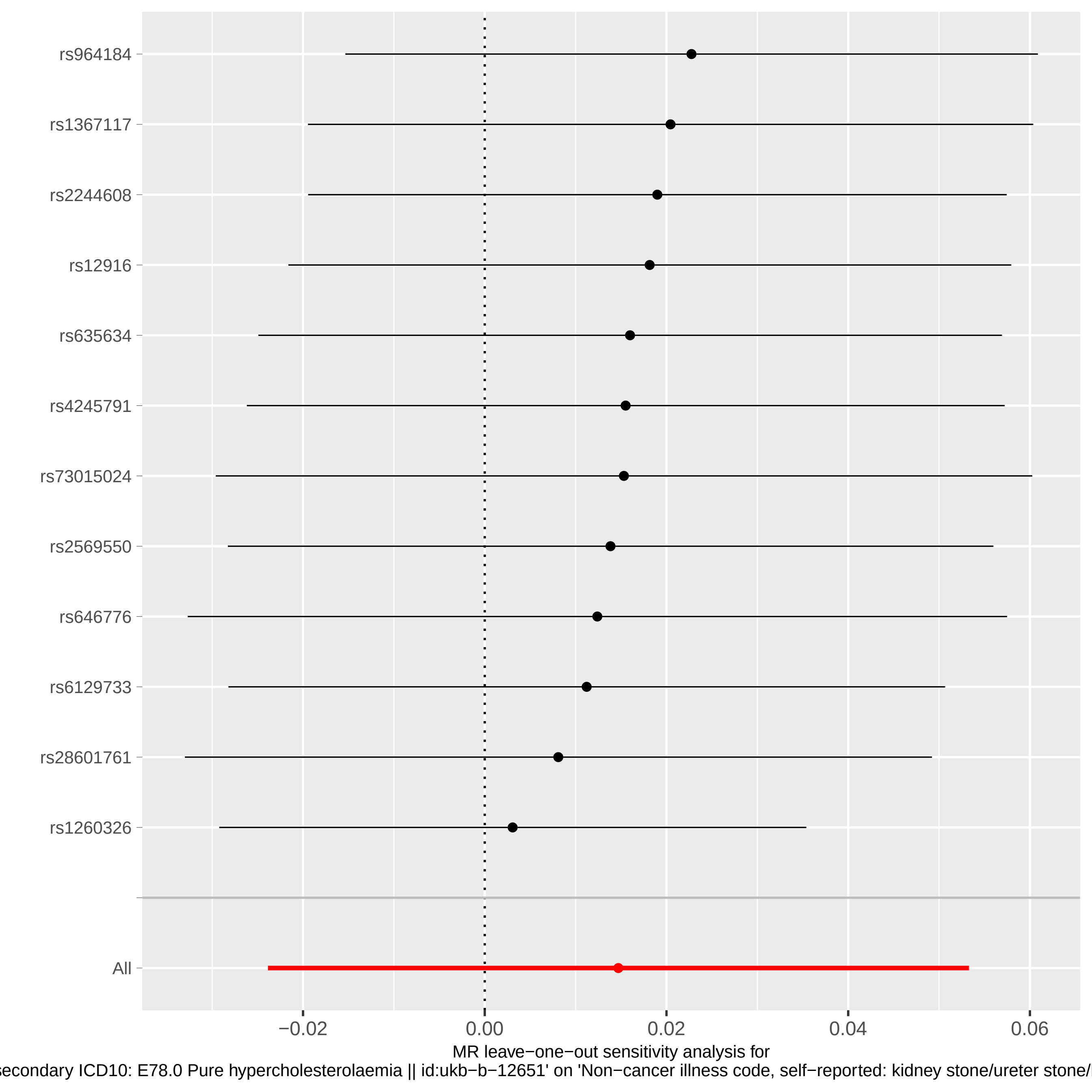
rs964184
rs1367117
rs2244608
rs12916
rs635634
rs4245791
rs73015024
rs2569550
rs646776
rs6129733
rs28601761
rs1260326
All
−0.02
0.00
0.02
0.04
0.06
MR leave−one−out sensitivity analysis for
secondary ICD10: E78.0 Pure hypercholesterolaemia || id:ukb−b−12651' on 'Non−cancer illness code, self−reported: kidney stone/ureter stone/

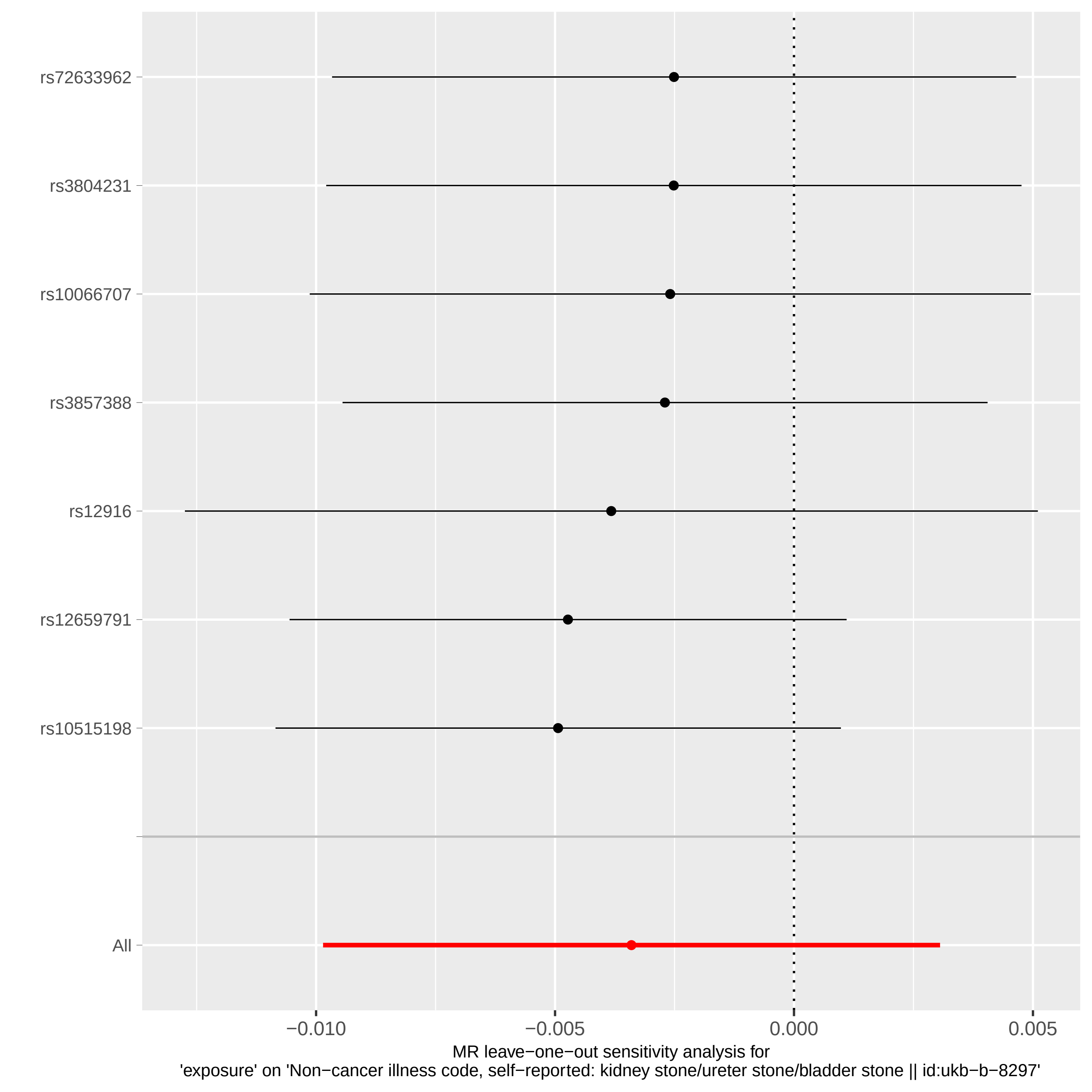
rs72633962
rs3804231
rs10066707
rs3857388
rs12916
rs12659791
rs10515198
All
−0.010
−0.005
0.000
0.005
MR leave−one−out sensitivity analysis for
'exposure' on 'Non−cancer illness code, self−reported: kidney stone/ureter stone/bladder stone || id:ukb−b−8297'

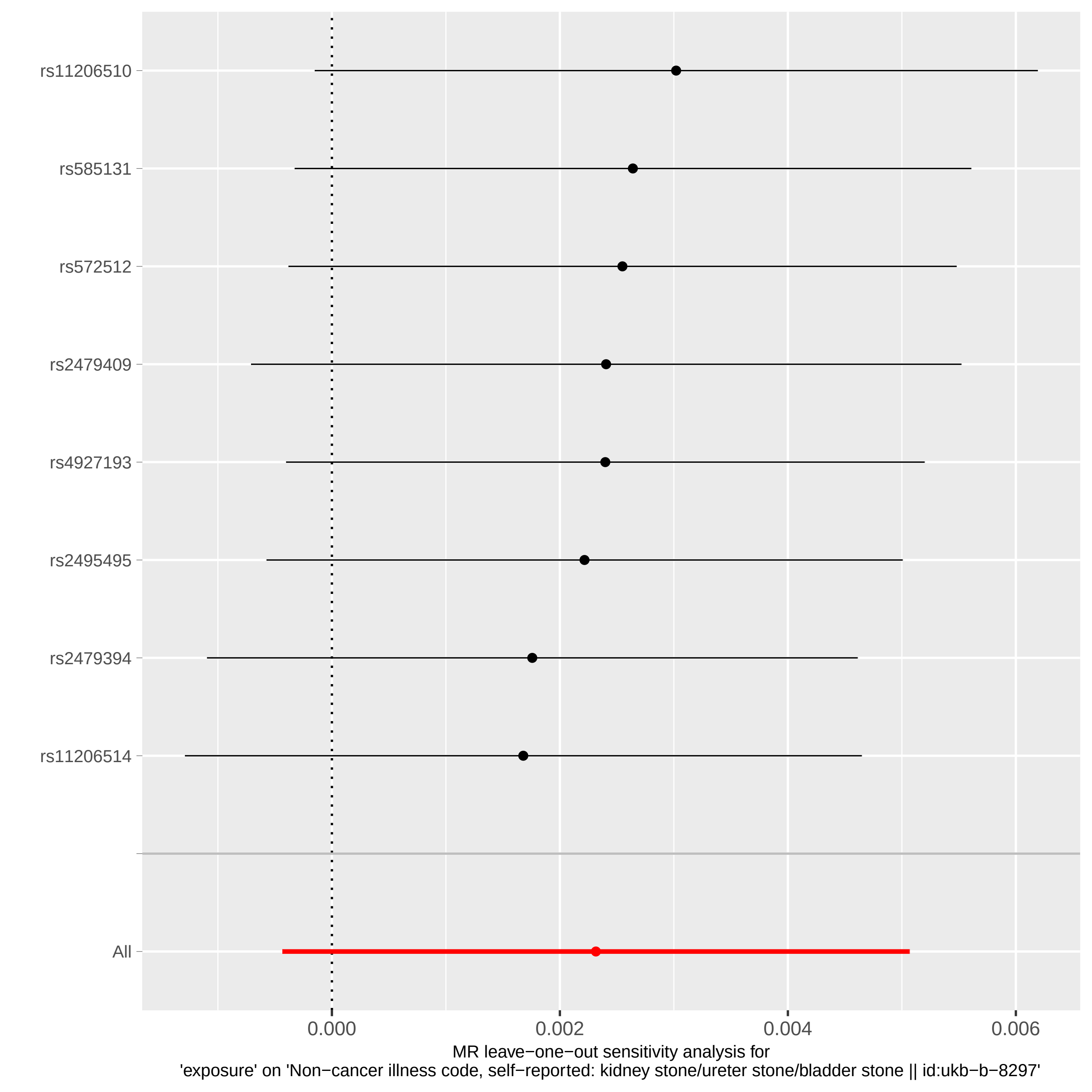
rs11206510
rs585131
rs572512
rs2479409
rs4927193
rs2495495
rs2479394
rs11206514
All
0.000
0.002
0.004
0.006
MR leave−one−out sensitivity analysis for
'exposure' on 'Non−cancer illness code, self−reported: kidney stone/ureter stone/bladder stone || id:ukb−b−8297'
