# Supplementary material for: Causal effects of circulating lipids and lipid-lowering drugs on the risk of urinary stones: a Mendelian randomization study
Source: Front Endocrinol (Lausanne). 2023 Dec 1;14:1301163. doi: 10.3389/fendo.2023.1301163 (PMC10722409; doi:10.3389/fendo.2023.1301163)
Supplement: Supplementary file 1 [file DataSheet_1.zip › Data Sheet 4.PDF]

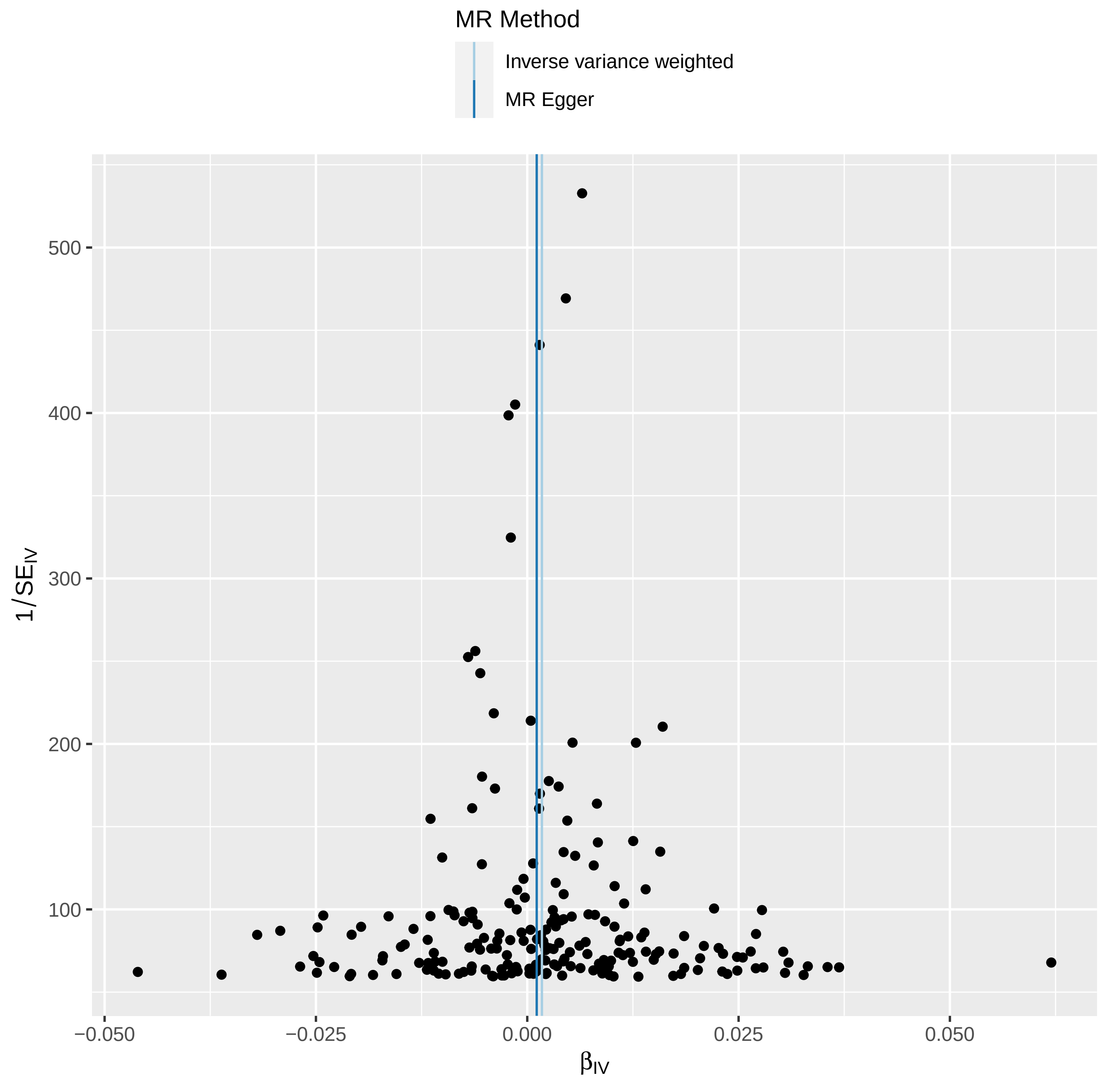

MR Method
Inverse variance weighted
MR Egger
500
400
300
200
100
$1/SE_{IV}$
−0.050
−0.025
0.000
0.025
0.050
β_{IV}

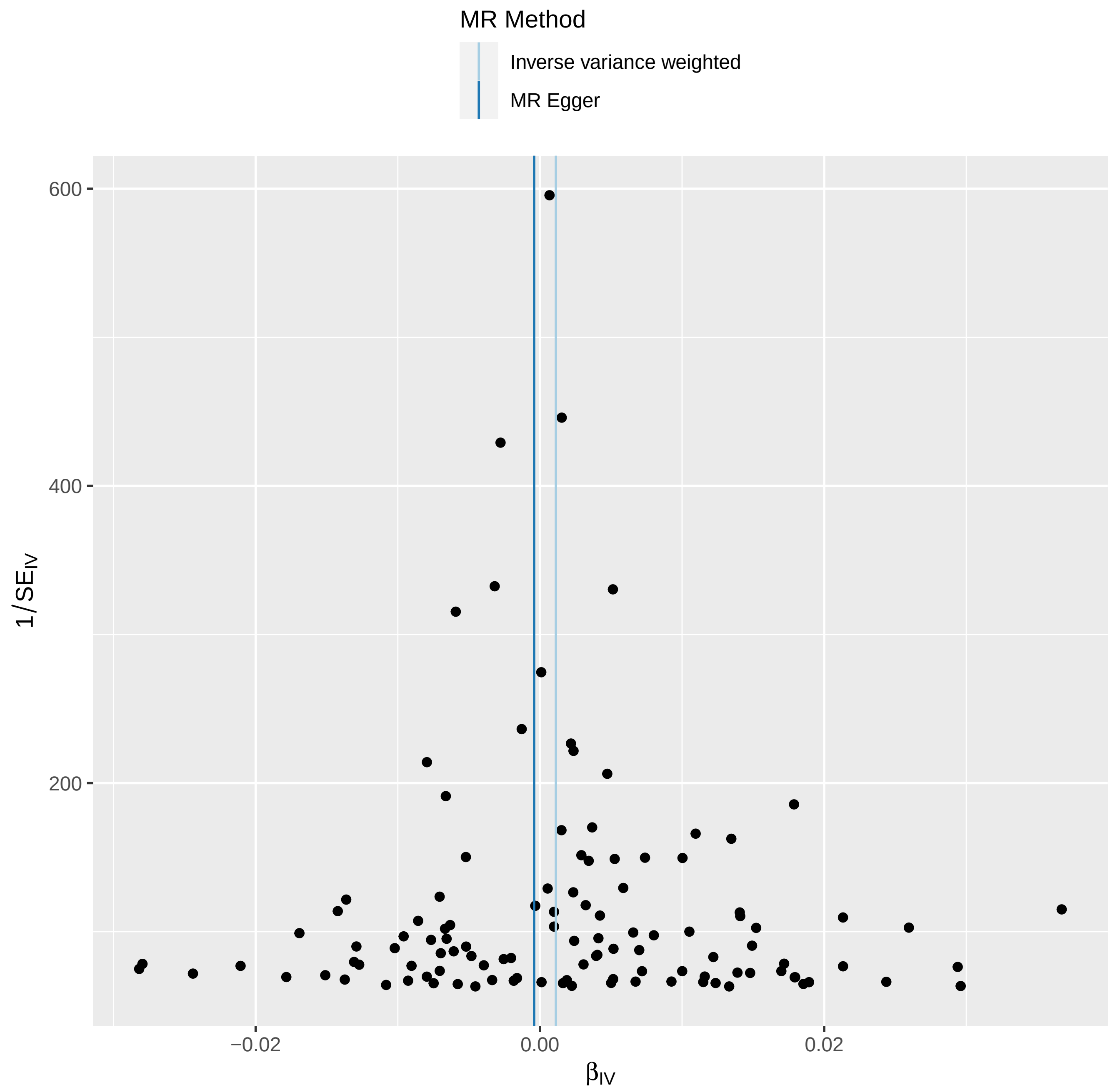

MR Method
Inverse variance weighted
MR Egger
600
400
200
1/SE_IV
−0.02
0.00
0.02
β_IV

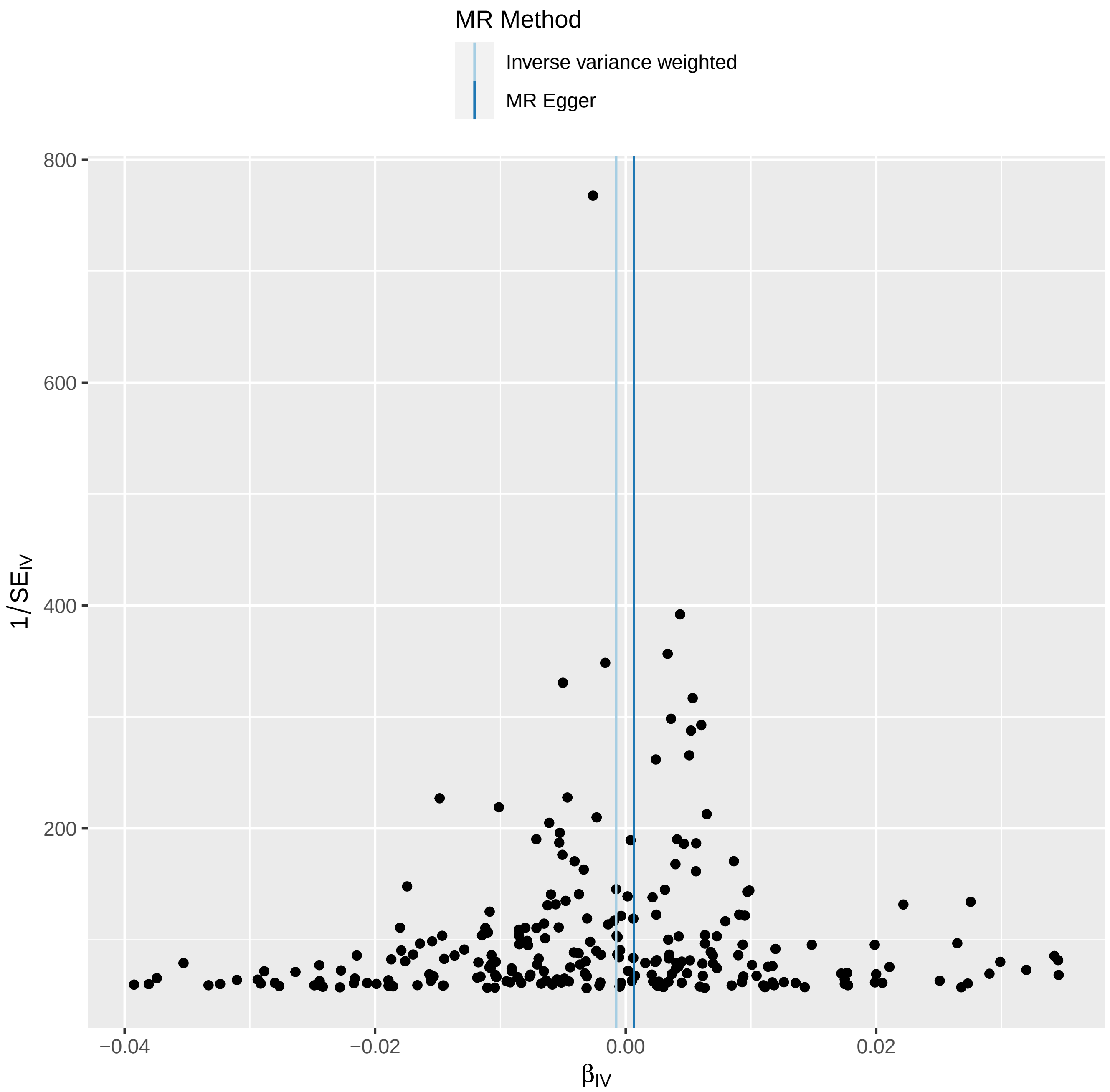
MR Method
Inverse variance weighted
MR Egger
800
600
400
200
$1/SE_{IV}$
−0.04
−0.02
0.00
0.02
β_{IV}

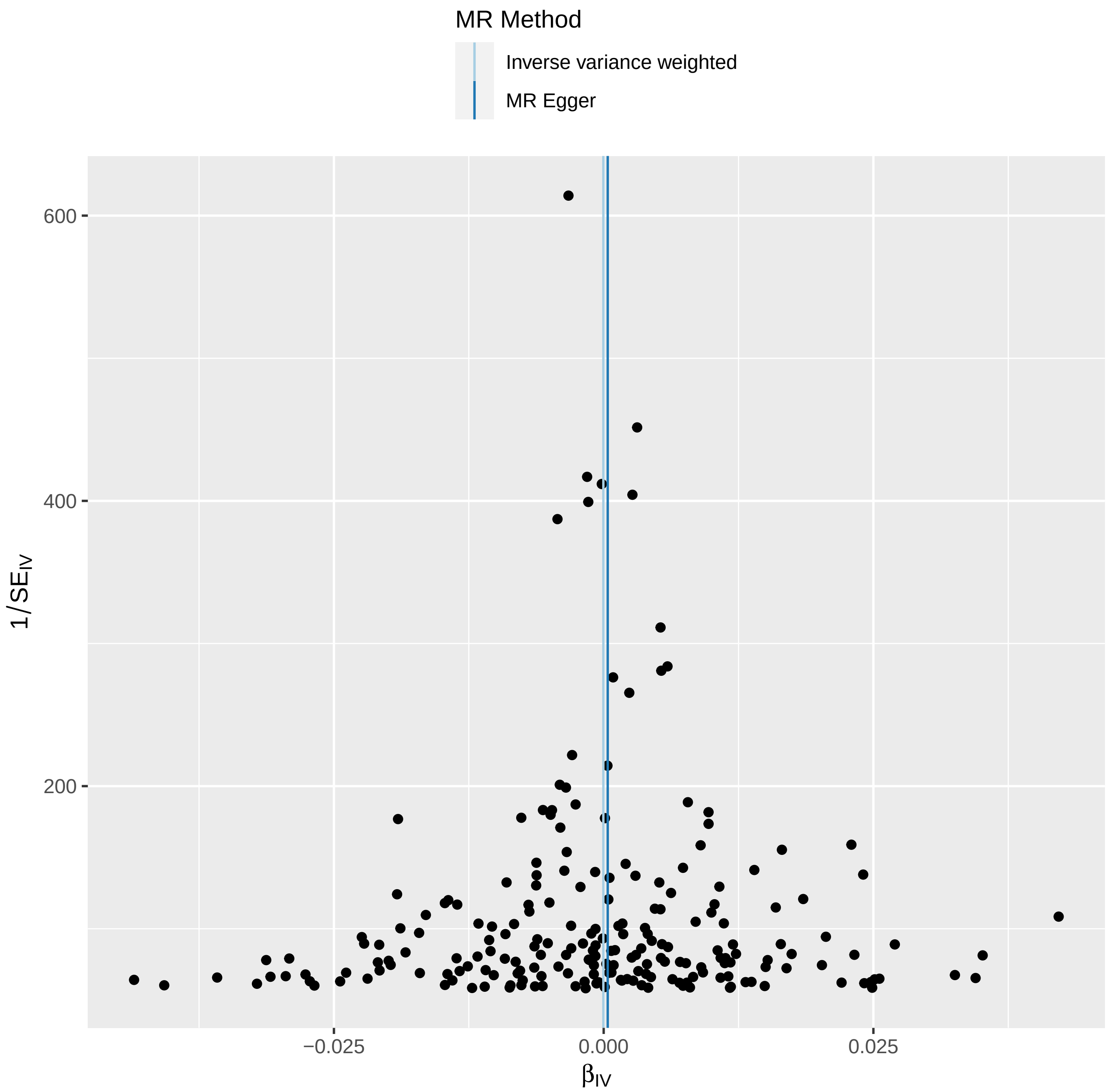
MR Method
Inverse variance weighted
MR Egger
600
400
200
$1/SE_{IV}$
−0.025
0.000
0.025
β_{IV}

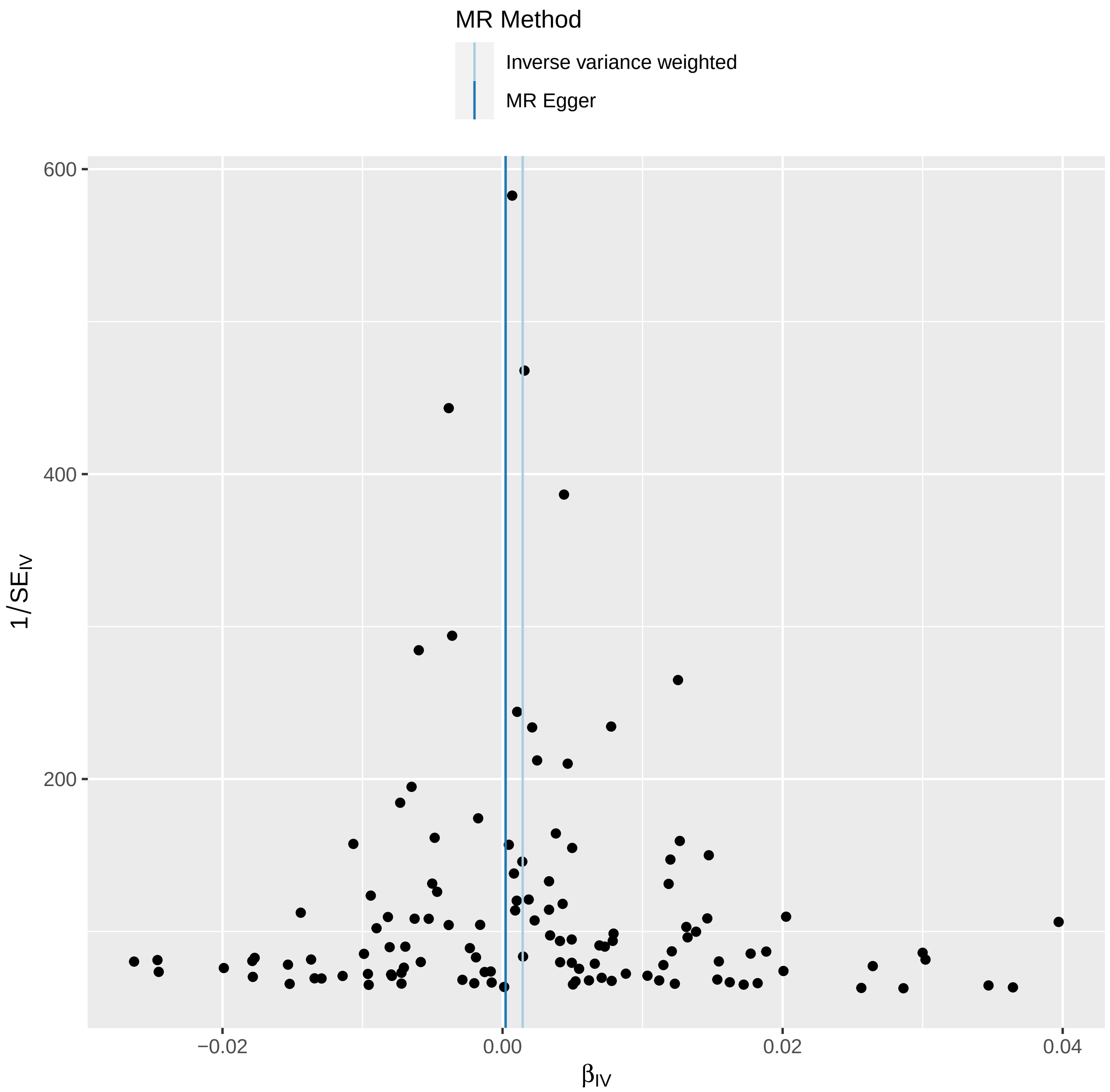

MR Method
Inverse variance weighted
MR Egger
600
400
200
1/SE_IV
−0.02
0.00
0.02
0.04
β_IV

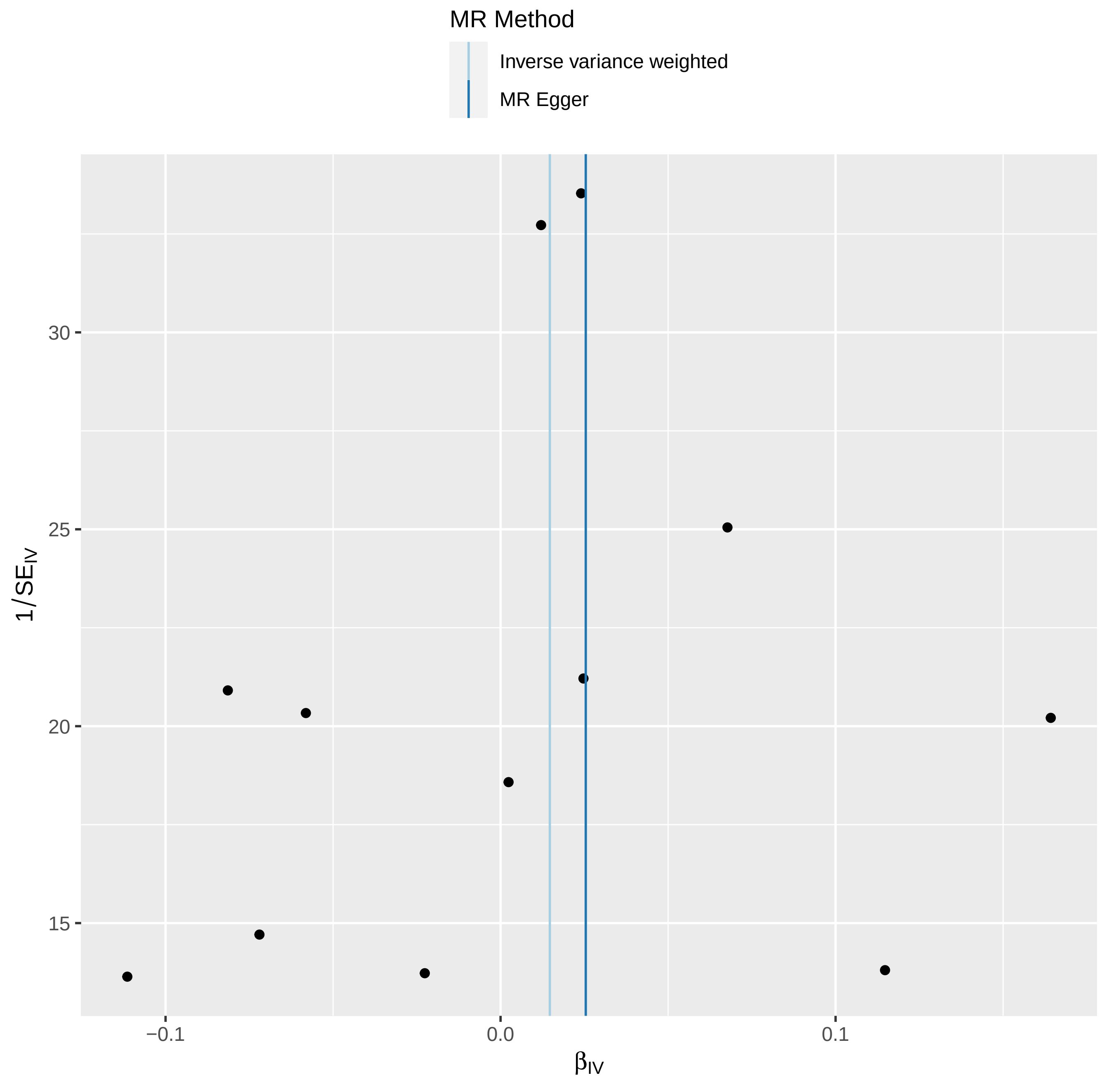

MR Method
Inverse variance weighted
MR Egger
30
25
20
15
1/SE$_{IV}$
−0.1
0.0
0.1
β$_{IV}$

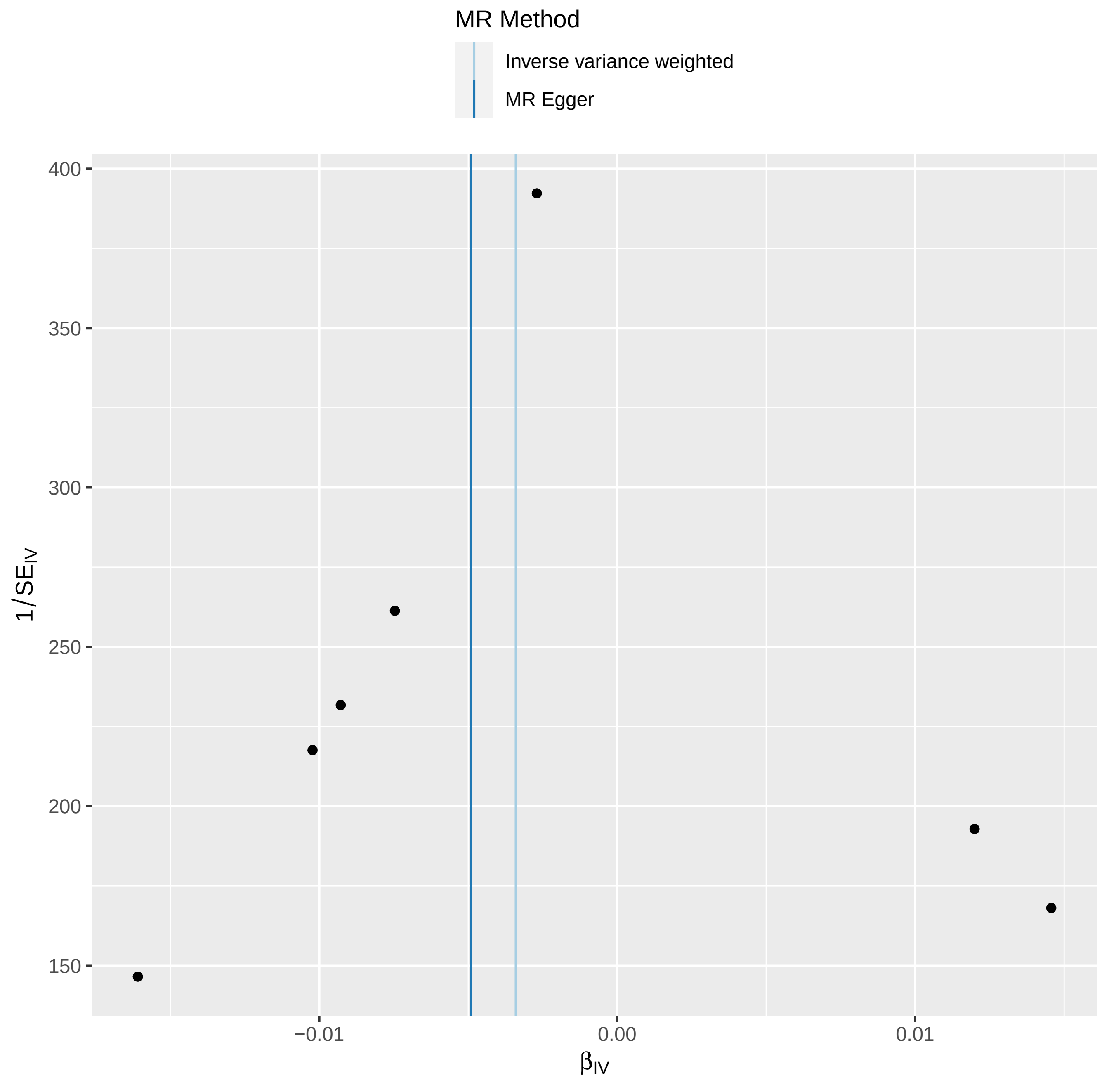
MR Method
Inverse variance weighted
MR Egger
400
350
300
250
200
150
$1/SE_{IV}$
−0.01
0.00
0.01
β_{IV}

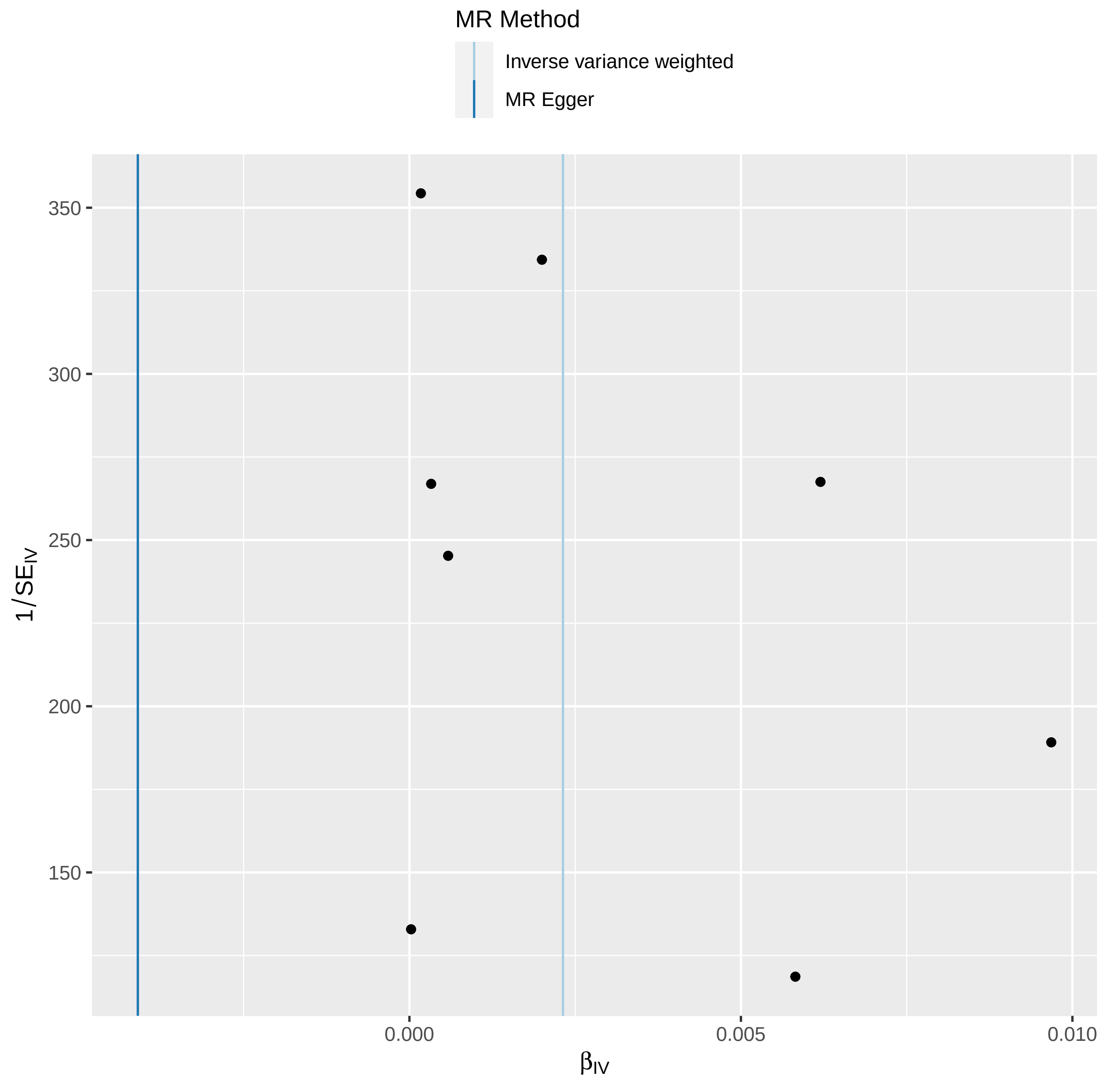

MR Method
Inverse variance weighted
MR Egger
350
300
250
200
150
1/SE_IV
0.000
0.005
0.010
β_IV
